# Development of a highly specific and sensitive VHH-based sandwich immunoassay for the detection of the SARS-CoV-2 nucleoprotein

**DOI:** 10.1016/j.jbc.2021.101290

**Published:** 2021-10-20

**Authors:** Marion Gransagne, Gabriel Aymé, Sébastien Brier, Gaëlle Chauveau-Le Friec, Véronique Meriaux, Mireille Nowakowski, François Dejardin, Sylvain Levallois, Guilherme Dias de Melo, Flora Donati, Matthieu Prot, Sébastien Brûlé, Bertrand Raynal, Jacques Bellalou, Pedro Goncalves, Xavier Montagutelli, James P. Di Santo, Françoise Lazarini, Patrick England, Stéphane Petres, Nicolas Escriou, Pierre Lafaye

**Affiliations:** 1 Département de Santé Globale, Institut Pasteur, Paris, France; 2 Plateforme d'Ingénierie des Anticorps, C2RT, Institut Pasteur, CNRS UMR 3528, Paris, France; 3 Plateforme technologique de RMN biologique, C2RT, Institut Pasteur, CNRS UMR 3528, Paris, France; 4 Plateforme Technologique Production et Purification de Protéines Recombinantes, C2RT, Institut Pasteur, CNRS UMR 3528, Paris, France; 5 Biology of Infection Unit, Institut Pasteur, Inserm U1117, Paris, France; 6 Lyssavirus Epidemiology and Neuropathology Unit, Institut Pasteur, Paris, France; 7 Molecular Genetics of RNA Viruses, UMR 3569 CNRS, University of Paris, Institut Pasteur, Paris, France; 8 National Reference Center for Respiratory Viruses, Institut Pasteur, Paris, France; 9 Evolutionary Genomics of RNA Viruses, Institut Pasteur, Paris, France; 10 Plateforme de Biophysique Moléculaire, C2RT, Institut Pasteur, CNRS UMR 3528, Paris, France; 11 Unité d'Immunité Innée, Institut Pasteur, Paris, France; 12 INSERM U1223, Paris, France; 13 Mouse Genetics Laboratory, Institut Pasteur, Paris, France; 14 Perception and Memory Unit, Institut Pasteur, CNRS UMR 3571, Paris, France

**Keywords:** antibody engineering, hydrogen-deuterium exchange, immunochemistry, phage display, single domain antibodies, nanobodies, COVID-19, nucleoprotein, diagnostic, CTD, C-terminal domain of the nucleoprotein, HDX-MS, hydrogen deuterium exchange–mass spectrometry, N, nucleoprotein, NTD, N-terminal domain of the nucleoprotein

## Abstract

The current COVID-19 pandemic illustrates the importance of obtaining reliable methods for the rapid detection of SARS-CoV-2. A highly specific and sensitive diagnostic test able to differentiate the SARS-CoV-2 virus from common human coronaviruses is therefore needed. Coronavirus nucleoprotein (N) localizes to the cytoplasm and the nucleolus and is required for viral RNA synthesis. N is the most abundant coronavirus protein, so it is of utmost importance to develop specific antibodies for its detection. In this study, we developed a sandwich immunoassay to recognize the SARS-CoV-2 N protein. We immunized one alpaca with recombinant SARS-CoV-2 N and constructed a large single variable domain on heavy chain (VHH) antibody library. After phage display selection, seven VHHs recognizing the full N protein were identified by ELISA. These VHHs did not recognize the nucleoproteins of the four common human coronaviruses. Hydrogen Deuterium eXchange–Mass Spectrometry (HDX-MS) analysis also showed that these VHHs mainly targeted conformational epitopes in either the C-terminal or the N-terminal domains. All VHHs were able to recognize SARS-CoV-2 in infected cells or on infected hamster tissues. Moreover, the VHHs could detect the SARS variants B.1.17/alpha, B.1.351/beta, and P1/gamma. We propose that this sandwich immunoassay could be applied to specifically detect the SARS-CoV-2 N in human nasal swabs.

Coronaviruses are a well-defined virus family that causes diseases in birds and mammals. To date, seven human coronaviruses have been identified. Common human coronaviruses, including types 229E, NL63 (both alpha coronaviruses), and OC43 and HKU1 (both beta coronaviruses) usually cause mild to moderate widespread illnesses such as the common cold (1). However, three severe epidemic events have also occurred in recent years caused by SARS-CoV-1, MERS-CoV, and SARS-CoV-2, three closely related coronaviruses. SARS-CoV-1 emerged in China in 2002–2003 and spread throughout this country. MERS-CoV caused an epidemic in 2012 that began in Saudi Arabia and was contained within the Middle East and Korea (2). SARS-CoV-2 was first isolated in December 2019 in Wuhan, China, and spread from China resulting in the global devastating COVID-19 pandemic (3).

Coronaviruses are enveloped viruses with a positive-sense RNA genome and a nucleocapsid of helical symmetry. Coronavirus nucleoproteins (N) localize to the cytoplasm and the nucleolus, a subnuclear structure, in both virus-infected primary cells and cells transfected with plasmids that express N. N is the most abundant coronavirus protein; it is required for coronavirus RNA synthesis and has an RNA chaperone activity that may be involved in template switch. N protein binds to the viral RNA during virion assembly, leading to the helical nucleocapsid formation (for a review, (4)).

The coronavirus Nucleoprotein is a homodimer formed by two monomers of about 48 kDa (5). Each monomer is composed of 2-folded domains respectively called the N-terminal domain (NTD) and the C-terminal domain (CTD). They are separated by a disordered region (called LKR), which could regulate the functions of N upon phosphorylation (6). NTD and CTD are both able to bind RNA (7, 8), while CTD also serves as a dimerization domain (9). Despite many studies, the mechanism by which the RNA genome is encapsidated by N has not been fully unraveled. Indeed, the structure of full-length N is not known probably due to the flexibility of the LKR region. The structures of the NTD and the CTD have only been determined by X-ray crystallography for MERS-CoV (10, 11) and very recently for SARS-CoV-2 (12). The interaction of the NTD with RNA has also been recently characterized by nuclear magnetic resonance (NMR) spectroscopy (13).

The current COVID-19 pandemic shows the importance of obtaining reliable solutions for the rapid and specific detection of SARS-CoV-2. Several solutions have been developed in record time. The reference method for diagnosis remains the RT-PCR (Reverse Transcriptase–Polymerase Chain Reaction), which allows the detection of SARS-CoV-2 RNA in either nasopharyngeal, salivary or pulmonary samples. This is an expensive method, which requires the transport of samples to well-equipped laboratories and their handling by qualified personnel. As the virus can only be detected in the body for a short time (typically 7–21 days after infection) (14), diagnostic tests should be performed as soon as the first symptoms appear or a contact is suspected. Therefore, diagnostic tests must be widely available and accessible, highlighting the importance of developing an antibody-based assay. Moreover, this assay must be specific and should not cross react with common human coronaviruses. As N is the most abundant protein of coronavirus (6), it is of upmost importance to develop diagnostic tests relying on antibodies that detect this protein.

Camelids produce two types of antibodies: (i) conventional antibodies made of dimers of heavy and light chains and (ii) a class of IgG devoid of light chain and made of dimers of heavy chains only (HC-IgGs) (15). The HC-IgGs comprise two antigen-binding domains (referred to as VHH or nanobodies). VHHs are the smallest available intact antigen-binding fragments with a molecular weight of around 15 kDa. They act as fully functional binding moieties and are readily produced in high amounts and active form in *E. coli.* Besides, they exhibit unique characteristics, such as enlarged complementarity determining regions (CDRs) and the substitution of three or four hydrophobic framework residues (which interact with the V_L_ in conventional antibodies) by more hydrophilic amino acids. Over the last decades, VHHs have progressively raised greater interest because of their specific properties. Indeed, they combine the high affinity and selectivity of conventional antibodies with the advantages of small proteins: in particular, they diffuse more readily into tissues owing to their small size and are able to reach intracellular antigens (16, 17, 18, 19, 20, 21), which allows them to be widely used for imaging.

VHHs have been raised against numerous viruses (reviewed in (22, 23)) including HIV (24, 25); influenza A (26, 27, 28); rabies virus (26); poliovirus (29); foot and mouth disease virus (30); Rotavirus (31), hepatitis C virus (32), and against SARS-CoV-1, MERS-CoV, and SARS-CoV-2 spike proteins (33, 34, 35, 36, 37). Recently VHHs specific of SARS-CoV-2 Nucleoprotein have been described (38). Although VHHs are monovalent, they frequently exhibit biological activities comparable to conventional bivalent antibodies (29). For instance, VHHs are able to bind to the SARS-CoV-2 spike protein and prevent infection of cells (33, 39).

Here we report the isolation and characterization of 7 VHHs directed against the N protein of SARS-CoV-2, which have been obtained by immunization of an alpaca with this nucleoprotein. These VHHs recognize specifically the SARS-CoV-2 N with nanomolar affinities and do not cross-react with common human coronaviruses. Their epitope has been mapped on either NTD or CTD by Hydrogen-Deuterium eXchange–Mass Spectrometry (HDX-MS). They are able to recognize the SARS-CoV-2 virus in infected cell cultures and pulmonary tissues from infected hamsters. An ELISA sandwich assay has been set up using VHH NTD E4-3 and VHH G9-1, allowing to detect as little as 4 ng/ml of N in solution. This ELISA sandwich is able to detect the nucleoprotein in human nasal swabs. These two VHHs can also detect the SARS-CoV-2 variants B.1.1.7/alpha, B.1-351/beta, and P1/gamma originally found in the United Kingdom, South Africa, and Brazil, respectively.

## Results

### Production and characterization of the recombinant full-length SARS-CoV-2 nucleoprotein

**Figure 1:**
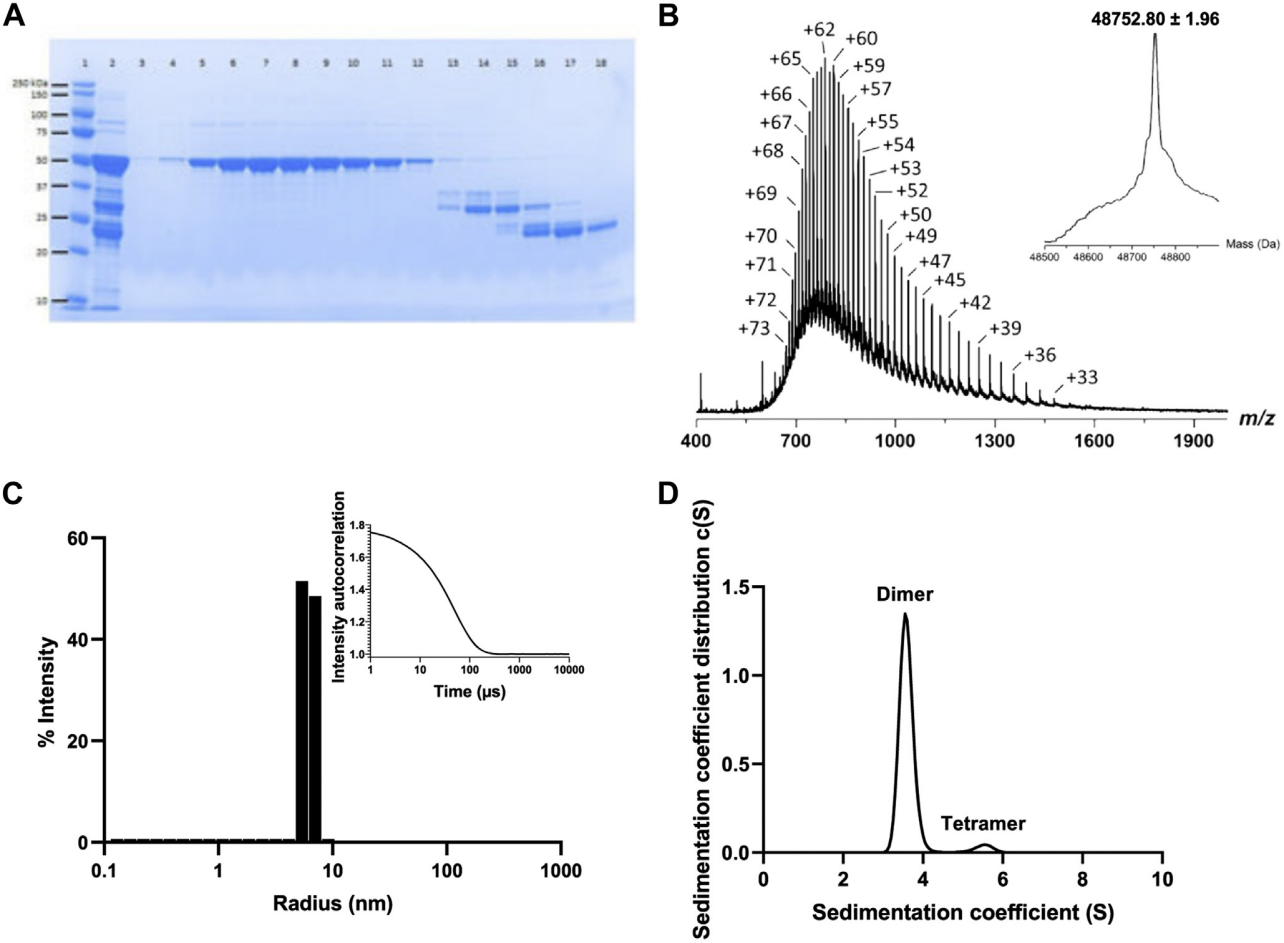
**Characterization of the Nucleoprotein**. *A* shows the SDS-PAGE gel with lanes 5–12 representing the eluted fractions containing the purified SARS-CoV-2 N and lanes 13–18 are separated contaminants; *B* represents the intact mass measurement. The measured molecular weight (48752.80 ± 1.96 Da) is consistent with the expected average mass calculated from the full-length SARS-CoV-2 N primary sequence (48752.13 Da, Δm = +0.67 Da (+13.7 ppm)), thereby confirming the structural integrity of the protein; *C* shows one main homogeneous population by DLS with a hydrodynamic radius of 6 nm. No aggregates are detectable at 37 °C; *D* represents the AUC measurement where 96% of the sample is under a dimeric form.

Recombinant full-length N was produced in *E.coli* (40) with a yield of 5.8 mg/g biomass and purified to homogeneity by affinity chromatography and gel filtration (Fig. 1*A*). The measured molecular weight (48752.80 ± 1.96 Da) was consistent with the expected mass of the protein (48752.13 Da), confirming the absence of degradation (Fig. 1*B*). Dynamic light scattering (DLS) experiments further showed that N is homogeneous and stable overnight at 37 °C and for at least 21 days at 4 °C (Fig. 1*C*). Finally, two distinct protein species were detected by sedimentation velocity analysis: the main one (96%) at a sedimentation coefficient of 3.6S compatible with a dimeric organization and the minor one (5%) at 5.5S compatible with a tetrameric organization (Fig. 1*D*). Altogether, these data reveal that N is stable at 4 °C and mainly forms a dimer in solution.

### Selection and characterization of VHHs recognizing N

An alpaca was immunized with the recombinant N protein and a library containing 5.85 × 10^7^ different VHHs was constructed from cDNA encoding VHH domains isolated from lymphocytes. VHHs were selected by phage display through three panning cycles against N, each with different buffer and washing conditions. In total, 400 individual clones were assayed by ELISA to test whether they could bind N. Five specific VHHs were thus obtained, called D12-3, E7-2, E10-3, G9-1, H3-3, respectively.

All of these VHHs recognized the CTD domain of N (see below). Therefore another panning was performed with NTD as bait, by using the same library and the same panning procedure. Two NTD-specific VHHs were thus isolated, called NTD E4-3 and NTD B6-1.

C-terminal strep-tagged VHHs were obtained by subcloning their genes into the pASK vector. The VHH production yields ranged from 0.1 mg/l to 1 mg/l of culture after Strep-Tactin affinity chromatography from periplasmic extracts. Quality control was performed showing that VHHs were not aggregated and had the expected molecular mass.

**Figure 2:**
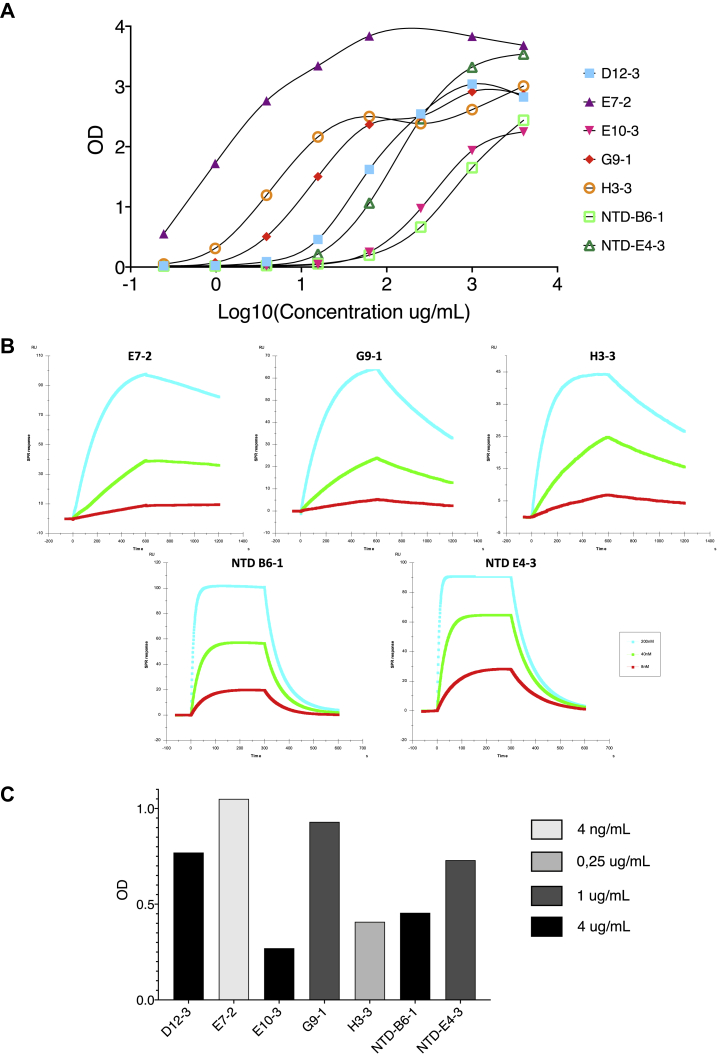
**Analysis of the binding of the different VHHs to the SARS-CoV-2 Nucleoprotein**. *A*, binding of the different VHHs to SARS-CoV-2 recombinant Nucleoprotein determined by ELISA. N was coated at 1 μg/ml and VHHs at different concentrations were then added. *B*, real-time monitoring of the VHH/N interaction by SPR. The determined kinetic parameters of the VHHS are provided in Table 1. *C*, binding of the VHHs by ELISA on cell extracts. The VHH concentration leading to maximal difference obtained between infected and uninfected cell extracts are indicated by *gray variations*.

The VHH binding activities were first analyzed by ELISA on the recombinant N. All the VHHs strongly bound to immobilized N (Fig. 2*A*), the highest signal was observed for E7-2 and the lowest for NTD B6-1. Surface plasmon resonance (SPR) studies showed that the VHHs bound to N with affinities in the nanomolar range (Table 1 and Fig. 2*B*). The measured KDs correlate with the ELISA results, with VHH E7-2 having the highest affinity (0.206 nM) and NTD B6-1 the lowest (46.5 nM).

**Table 1:** Kinetic parameters of the interaction between the SARS-CoV-2 nucleoprotein and the different VHHs

VHH	K_d_ (nM)	k_on_ (10^5^ M^−1^ s^−1^)	k_off_ (10^−4^ s^−1^)
D12-3	5.78 ± 1.15	13.8 ± 3.1	77.2 ± 2.1
E7-2	0.206 ± 0.073	7.05 ± 2.04	1.81 ± 0.56
E10-3	24.1 ± 4.0	1.49 ± 0.26	35.5 ± 7.0
G9-1	2.40 ± 0.38	5.96 ± 1.27	14.0 ± 1.4
H3-3	0.902 ± 0.119	10.0 ± 0.9	9.00 ± 1.09
NTD B6-1	46.5 ± 2.6	4.24 ± 0.57	201 ± 29
NTD E4-3	23.8 ± 2.1	5.97 ± 0.94	140 ± 11

An ELISA was then performed on infected and uninfected cell extracts (Fig. 2*C*), showing that all the VHHs recognized the N present in infected cell extracts. The different color bars represent the concentration of VHH for which the maximal OD difference between infected and uninfected extracts was obtained. VHHs can be classified in different groups: E7-2 shows the best signal at a concentration as low as 4 ng/ml; followed by VHH H3-3 at 0.25 μg/ml, VHHs G9-1 and NTD-E4-3 at 1 μg/ml, and finally E10-3, D12-3, and NTD-B6-1 at 4 μg/ml.

The specificity of the VHHs for SARS-CoV-2 N was assessed in an ELISA, by comparing their binding to the seasonal human coronaviruses (OC43, HKU1, 229E, and NL63), with that to SARS-CoV-1 and SARS-CoV-2, using the SARS-CoV-2 spike protein as a negative control. We did not observe any binding to the N of seasonal coronaviruses. VHHs NTD-E4-3, D12-3, and E10-3 recognized SARS-CoV-2 N better than SARS-CoV-1 N. Nevertheless VHH E7-2 interacted with the N of seasonal coronavirus as well as with the spike protein but to a lesser extent than for SARS-CoV-1 and SARS-CoV-2. This non-specific binding might be explained by a local denaturation of N when coated on ELISA plates.

#### Identification of the SARS-CoV-2 N antigenic regions recognized by each VHH

SPR experiments were first performed to determine if the VHHs recognized overlapping or distinct epitopes on N. VHHs E7-2, G9-1, and H3-3 recognized overlapping epitopes while NTD B6-1 and NTD E4-3 recognized a different epitope.

HDX-MS was used to locate more precisely the binding sites of each VHH on N. The quench and pepsin conditions were first optimized to generate a peptide map with high sequence coverage and peptide redundancy. A total of 51 unique peptides covering 94.4 % of the N sequence were selected and brought to the HDX-MS analysis.

**Figure 3:**
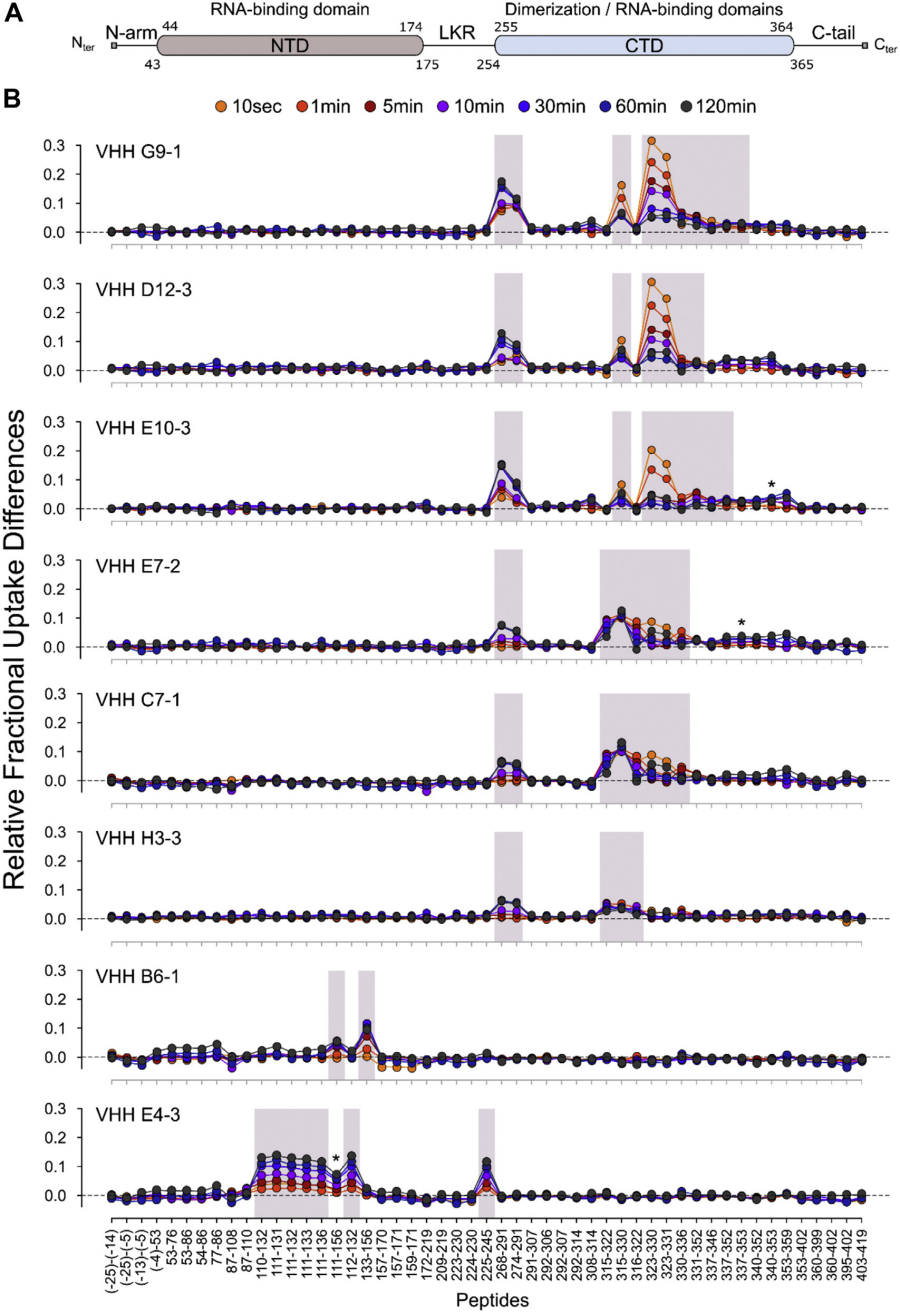
**Identification of the VHH binding sites by HDX-MS**. *A*, general organization of full-length SARS-CoV-2 N showing the position of the 2-folded NTD and CTD structural domains and the three intrinsically disordered regions (N-arm, LKR, and C-tail). *B*, differential fractional uptake plots showing the relative variations in deuterium incorporation imposed by the binding of each VHH to full-length SARS-CoV-2 N. The differences in uptake between the apo- and VHH-bound states were calculated for each peptide and time point and plotted as a function of peptide position. A positive uptake difference is indicative of a VHH-induced protective effect on the exchangeable amide hydrogens. Peptides displaying statistically significant uptake differences (Wald test, *p* < 0.01) are highlighted in *gray*. Peptides 340–353 (panel VHH E10-3), 337–353 (panel VHH E7-2), and 111–156 (panel VHH E4-3) were removed from the statistical analysis due to either poor fitting quality to the Mixed Effect Model or poor MS signal.

Epitope mapping was performed by comparing the deuterium exchange profiles of N in its apo- and VHH-bound states. The relative fractional uptake difference plots obtained for each VHH are presented in Figure 3. A positive uptake difference value indicates a VHH-induced protective effect on the exchangeable amide hydrogens. As reported in Figure 3*B*, VHHs G9-1, D12-3, E10-3, E7-2, and H3-3 all targeted the CTD domain and reduced the solvent accessibility of similar peptides encompassing regions 268-291 and 316-330. Inspection of the different overlapping peptides revealed that the main reductions of solvent accessibility occurred in segments 268-273 and 323-330 for D12-3, E10-3, and G9-1 and in segments 268-291 and 316-322 for H3-3. All these VHHs therefore share a common binding interface made of two distal regions in the CTD primary structure (*i.e.*, conformational epitopes).

The two VHHs selected against NTD, namely NTD B6-1 and NTD E4-3, interacted with distinct and nonoverlapping epitopes. As shown in Figure 3*B*, VHH NTD B6-1 targeted a linear epitope containing residues 137–156 *only*. The binding of VHH NTD E4-3 on the other hand affected the solvent accessibility of two regions encompassing residues 110–156 of the NTD domain and residues 225–245 of the intrinsically disordered LKR linker (Fig. 3*B*). These two regions contained several overlapping peptides with similar back-exchange values (Table S1) allowing to confine the effect of NTD E4-3 to segments 111–133 and 231–245 *only*. This finding was unexpected since VHH NTD E4-3 was selected using recombinant NTD as a bait. In the absence of crystal structure of full-length SARS-CoV-2 N, the variations observed in LKR may result either from: (1) a direct masking effect imposed by the VHH; in this scenario NTD E4-3 targets a conformational epitope containing a dominant sequential element located in the NTD domain; or (2) from steric hindrance and/or allosteric changes induced upon E4-3 binding; in this scenario, the NTD E4-3 epitope is linear and only formed by segment 111–133.

**Figure 4:**
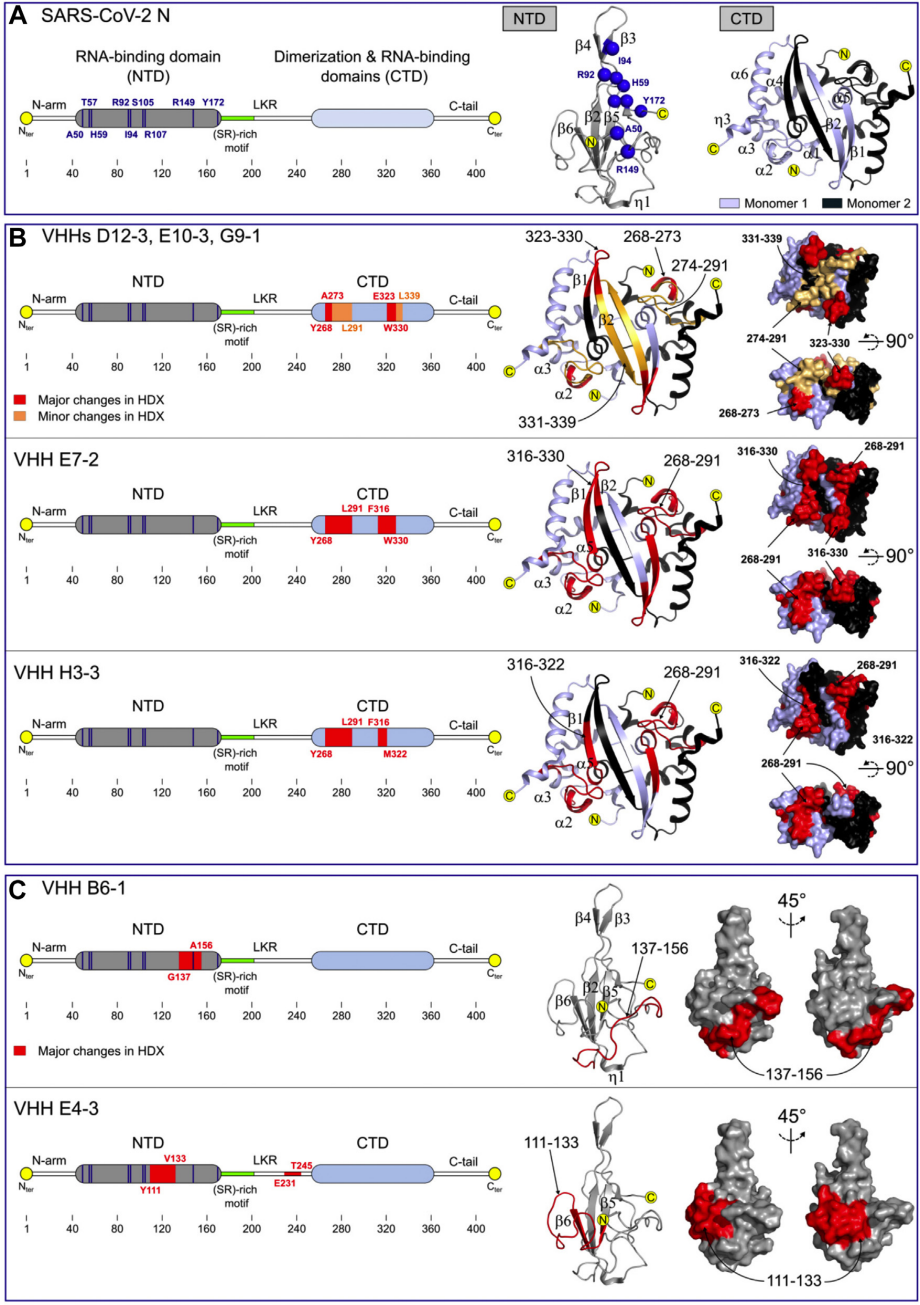
**Comparison of the VHH epitopes mapped by HDX-MS**. *A*, linear representation (*left*) of full-length SARS-CoV-2 N with NTD in *gray* and CTD in *light blue*. The cartoon representations of the NTD domain (pdb # 7CDZ) and the CTD dimer (pdb # 7CE0) are shown on the *right panel*, with one CTD monomer colored *light blue* and the other colored *black*. The RNA-binding residues identified by NMR are also reported on the linear (*left*, *blue bars*) and the cartoon representations of the NTD domain (*right*, *blue spheres*). *B* and *C*, comparison of the epitopes identified in the CTD (*B*) and the NTD (*C*) domains by HDX-MS. Red and orange patches correspond to regions were major and minor reductions in solvent accessibility were observed upon VHH binding. HDX-MS results are mapped onto the cartoon and surface representations of the NTD and CTD domains.

To better visualize and compare the position of each epitope, HDX-MS results were mapped onto the crystal structures of SARS-CoV-2 NTD and CTD (Fig. 4). As shown in Figure 4*B*, the HDX-MS defined epitopes recognized by G9-1, D12-3, E10-3, E7-2, and H3-3 in the CTD domain are conformational and contained the long and solvent exposed loop connecting helices α2 to α3 (region 268–291). The only differences were observed within region 316–339. Whereas E7-2 affected the entire 316–330 region (α5-β1-β2), the main changes in solvent accessibility were restricted to segment 323–330 (β1-β2) for D12-3, E10-3, and G9-1 and to segment 316–322 (α5-β1) for H3-3. Our results also revealed that VHH NTD B6-1 targeted a linear epitope formed by the long and solvent exposed C-terminal NTD loop (Fig. 4*C*). Finally, in the absence of full-length N structure, the type of epitope targeted by VHH E4-3 remains ambiguous. Nevertheless, our results reveal that the E4-3 epitope is made of the long NTD loop connecting β5 to β6 (Fig. 4*C*).

Altogether, these experiments revealed that VHHs G9-1, D12-3, E10-3, E7-2, and H3-3 specifically targeted and shared a common binding site on CTD, whereas VHHs E4-3 and B6-1 both recognized distinct and nonoverlapping epitopes on the NTD domain.

#### Recognition of infected cells by Immunofluorescence

**Figure 5:**
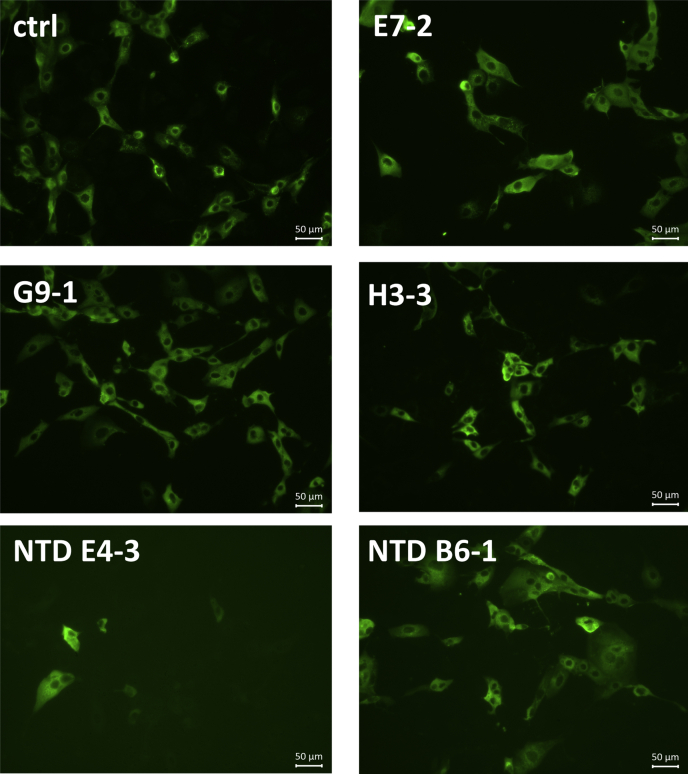
**Immunofluorescence labeling of SARS-CoV-2 virus in infected cells**. Representative staining with biotinylated VHHs at 1 μg/ml of subconfluent layer of FRhK4 infected cells. A rabbit antibody against the SARS-CoV-2 N was used as a control of the infection.

FRhK4 cells were infected with the SARS-CoV-2 virus. After 24 h, the subconfluent layer of cells was fixed and permeabilized. A control with rabbit polyclonal antibodies against SARS-CoV-2 N labeled with an anti-rabbit Alexa Fluor 488 allowed us to evaluate that around 50% of cells were infected. Biotinylated VHHs were used at a concentration of 1 μg/ml and labeled with streptavidin Alexa-Fluor 488. All the fluorescent VHHs labeled the infected cells, as shown in Figure 5, whereas no labeling was observed on uninfected cells (data not shown), suggesting that they all recognized the SARS-CoV-2 virus *in situ*. The exposition for imaging needed to be adjusted for each VHH. These variabilities in sensitivity are consistent with the difference of affinities observed between the VHHs.

#### Recognition of SARS-CoV-2 virus in infected hamster tissues

**Figure 6:**
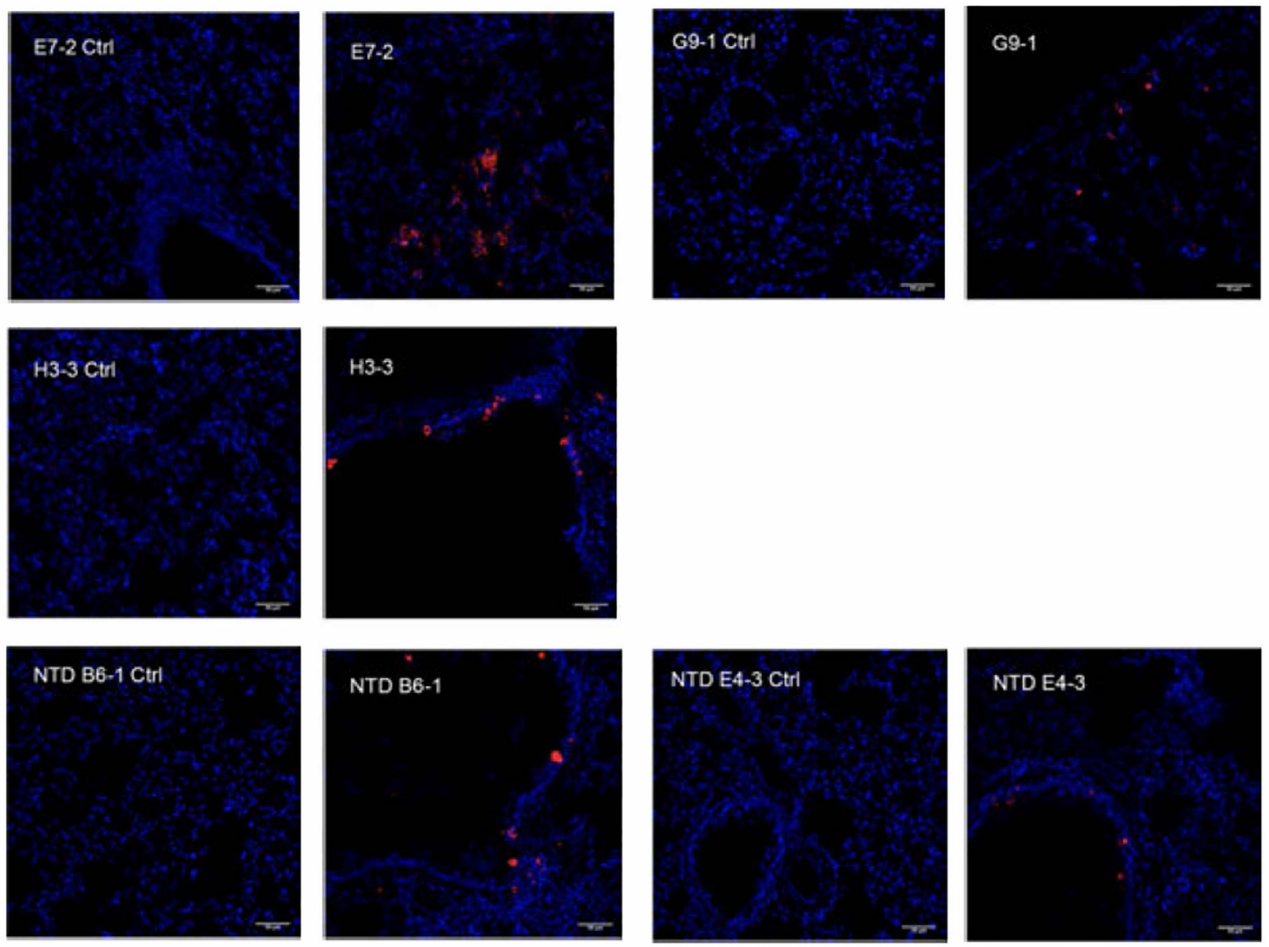
**Immunofluorescence labeling of SARS-CoV-2 virus in the lung of infected Syrian Hamster.** Representative staining of lung slices with biotinylated VHHs at 1/500.Scales bar: 50 μm. *Left panel*, uninfected control; *right panel*, infected hamster

Syrian hamsters were infected with the SARS-CoV-2 virus (41). The lungs were recovered and fixed and unstained sections were incubated with 2 μg/ml of each VHH. A control was performed with uninfected hamster lungs. A strong labeling of virus present in bronchoalveolar epithelium was observed with the different biotinylated VHHs used (E7-2, G9-1, H3-3, NTD B6-1, and NTD E4-3), representative of the different epitopes targeted (Fig. 6). However, significant variations of labeling intensity were observed between the different VHHs. Even if we cannot exclude that these could be due to the differences in affinity of the VHHs or to the accessibility of their epitopes, the most likely explanation is that the number of infected cells varied somewhat between different sections of lung tissue. Each VHH was indeed incubated within a different section of the lung, and although all sections were adjacent in the tissue, the size of the foci of infection varied from one section to another.

#### Detection of nucleoprotein in solution by sandwich ELISA

A sandwich ELISA was set up to detect in solution the full-length SARS-CoV-2 N. The three best VHHs against CTD (E7-2, G9-1, and H3-3) were used in combination with the two anti-NTD VHHs (NTD-B6-1 and NTD-E4-3) to determine which was the optimal couple for the detection of N. VHH G9-1 and NTD-E4-3 gave respectively the best signals among anti-CTD and the anti-NTD VHHs. As little as 4 ng/ml of SARS-CoV-2 N could be detected. The sandwich E4-3/G9-1 ELISA was specific of SARS-CoV-2 N because no detection of seasonal human coronaviruses N was observed (data not shown).

The VHHs were also able to detect inactivated and permeabilized SARS-CoV-2 virus. The same ranking was observed: NTD-E4-3 in combination with the CTD G9-1 was the best couple to detect the SARS-CoV-2 N in native conditions.

SARS-CoV-2 replicates mainly in the human upper respiratory tract (42, 43). Therefore, human nasopharyngeal swabs from healthy controls and COVID-19 patients were tested using the sandwich E4-3/G9-1 ELISA. The results from the sandwich ELISA were compared with an available commercial immunochromatographic assay. The immunochromatographic assay can detect as little as 1 ng/ml of N. Quantification of N by ELISA was made possible by using a calibration curve. Five samples were considered as negative by PCR (#1, #2, #3, #6, #79) and 12 positive (#4, #5, #7, #12, #14, #22, #30, #45, #47, #58, #60, #64, #67). We observed a good correlation between the two techniques (Table 2). N could not be detected in samples #1, #2, and #3 either by the immunochromatographic assay or by ELISA. However, N was detected in sample #6 (healthy control, PCR neg) using the immunochromatographic assay but not by the VHH ELISA. On the other hand, the ELISA detected N in sample #79 even if it was considered as negative by PCR (39 days postinfection). As for PCR-positive samples, N could not be detected in samples #14, #22, and #30 by ELISA or the immunochromatographic assay. For sample #60, a band was detected by the immunochromatographic assay but not by the ELISA.

**Table 2:** Comparison of the detection of nucleoprotein in human nasal swabs by an immunochromatographic assay and a sandwich ELISA

Samples	Immunochromatographic assay	Sandwich ELISA (ng/ml)
#1	−	0
#2	−	0
#3	−	5
#4	+	45
#5	+	39
#6	+/−	0
#7	+	15
#12	+	24
#14	−	0
#22	−	0
#30	−	0
#45	+	0
#47	+	34
#58	+	117
#60	+	0
#64	+	16
#67	+	29
#79	+	38

#### Detection of SARS-CoV-2 variants of concern

Several variants have emerged recently B.1.1.7/alpha, B.1.351/beta, and P1/gamma and have spread in multiple countries due to increased transmission (44, 45). These variants of concern harbor mutations in the spike but also in the N protein, which could affect their detection in antibody-based tests. Therefore, we analyzed the binding of VHH G9-1 and VHH NTD E4-3 on these variants.

**Figure 7:**
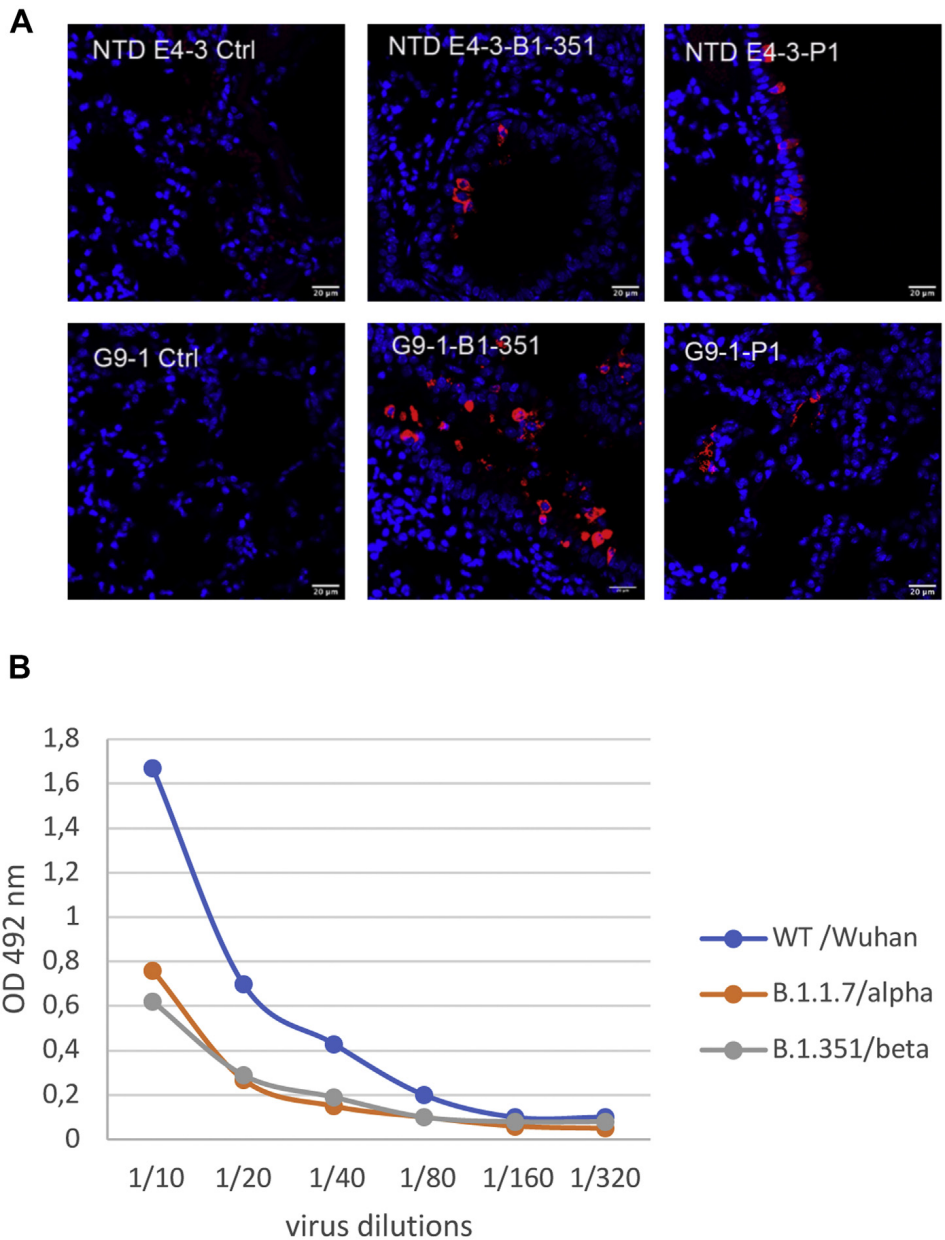
**Detection of SARS-CoV-2 variants of concern by VHHs.***A*, immunofluorescence labeling of N protein in lung sections of mice infected with the B.1-351 and P1 SARS-Cov-2 variants. Representative staining of lung slices with biotinylated VHHs at 1/500.Scales bar: 50 μm. *Left panels*, uninfected control; *right panels*, infected mice. *B*, detection of Nucleoprotein from variants by sandwich ELISA. VHH NTD-E4-3 was coated on the plate, permeabilized SARS-CoV-2 virus variants were then added at different concentrations and were revealed by adding a biotinylated VHH G9-1 followed by peroxydase labeled streptavidin. Controls without virus were performed and their values were substracted from the data.

We tested the ability of VHHs NTD E4-3 and G9-1 to detect the N protein on fixed tissues. Mice were infected with the B.1.351 and P1 variants as described in (46). Sections of formalin-fixed lungs were incubated with 2 μg/ml of biotinylated VHHs NTD E4-3 and G9-1. Uninfected mouse lung was used as control. Strong labeling was observed with both VHHs in mice infected with either variants (Fig. 7*A*).

We also performed a sandwich immunoassay on uninfected and infected cell extracts using Wuhan, B.1.1.7, and B.1.351 variants (Fig. 7*B*) and found that both VHHs NTD E4-3 and G9-1 recognized the N protein in all infected cell extracts.

## Discussion

We have obtained seven different VHHs that recognize the SARS-CoV-2 Nucleoprotein. N was expressed in *E. coli* as a dimer. By HDX-MS we have confirmed that the NTD and CTD regions are structured unlike the N-arm, the LKR region, and the C tail. N was then used for immunization of an alpaca. The first VHHs isolated after panning with the whole protein were all directed against CTD. Another panning against NTD was required to isolate VHHs specific of this domain. This bias might be due either to the fact that N coated on polystyrene tubes could expose CTD preferentially or that VHHs against NTD could be counter-selected because of their lower affinity. VHHs directed against CTD (E7-2, H3-3, G9-1, E10-3, and D12-3) recognized overlapping epitopes and SPR experiments showed that these VHHs were unable to bind simultaneously, suggesting the existence of an immunodominant epitopic region in the CTD. The two anti-NTD VHHs recognized different epitopes.

The different VHHs recognized N in infected cells and in infected hamster or mice tissues showing their ability to recognize the native nucleoprotein. The development of a sandwich ELISA also allowed the detection of native N. These data suggest that the presence of RNA on N does not seem to impede the VHH binding.

We tested all the VHHs on nucleoproteins from different human coronaviruses. They recognized N from SARS-CoV-1 and SARS-CoV-2 but not from other coronaviruses. The protein sequences of the SARS-CoV-1 and SARS-CoV-2 nucleoproteins have a 90% identity, while the other nucleoproteins only have a 28–33% identity. The sequence of the epitopic regions recognized by the seven VHHs is different between the common human and SARS coronaviruses explaining the specificity of our VHHs for SARS N. As SARS-CoV-1 virus is not circulating anymore, VHHs are compelling to set up a specific detection test.

Recently, several variants emerged (44, 45), which are more efficiently transmitted. They present several mutations mostly in the Spike protein but also in the Nucleoprotein. The N mutations are D3L and S235F for the variant B.1.1.7/alpha, T205I for the variant B.1.351/beta and P80R for the variant P1/gamma (47). Interestingly most of these mutations occur either in the N terminal arm (position 3) or in the LKR (positions 205 and 235) two intrinsically disordered regions. A mutation is also observed at position 80 at the N terminal end of NTD close to the N arm. We have shown that the sandwich ELISA NTD E4-3/G9-1 recognized N in B.1.1.7 and B.1.351 permeabilized cells, and a strong labeling was observed with both VHHs in mice infected with B.1.351 and P1 variants showing their ability to recognize the native N present in the different variants. These data suggest that our VHHs target conserved regions not prone to mutations, which is of utmost importance for the robustness of a diagnostic test.

Interestingly, the VHH NTD E4-3 recognized preferentially SARS-CoV-2 N. The epitope as defined by HDX-MS was ambiguous with the involvement of segments 111–133 and 231–245 even if this VHH was selected using recombinant NTD (segment 1–200) as a bait. Three aa differences are observed in region 111–133: in position 120 a glycine is present for SARS-CoV-2 while it is a serine for SARS-CoV-1, an aspartic acid instead of a glutamic acid in position 128 and an isoleucine is in place of a valine in position 131. Aspartic acid and glutamic acid are quite similar amino acid, and we might suggest that positions 120 and 131 are important for the binding of VHH NTD E4-3. The HDX-defined NTD B6-1 epitope contained the positively charged R149 residue recently identified as important for RNA binding (Fig. 4, *A* and *C*) (13). Among the regions recognized by the anti CTD VHHs, only one difference at position 290 (aspartic acid instead of glutamic acid) is observed between the two SARS nucleoproteins. This position is probably not involved in the binding as some anti CTD VHHs present the same binding for both nucleoproteins.

We determined the best combination of VHHs to detect the nucleoprotein first as a recombinant protein, then on a permeabilized virus. We found that coating the anti-NTD E4-3 for the capture and using the anti-CTD G9-1 to reveal the nucleoprotein is the best option. No cross-reaction was observed with other human seasonal coronaviruses N due to the exquisite specificity of both VHHs. Moreover the alpha, beta, and gamma variants were detected by these two VHHs highlighting the wide recognition efficiency of this sandwich immunoassay. Recently Anderson *et al.* (38) have described VHHs directed SARS-CoV-2 Nucleoprotein. They have developed a sensitive sandwich immunoassay, but there is no indication if these VHHs recognized or not the native N, the seasonal coronaviruses, and the alpha, beta, and gamma variants.

This assay has been used to test the presence of N in human nasal swabs. In parallel an immunochromatographic assay (“Rapid SARS-CoV-2 Antigen test Card”) was used. Both tests can detect low amounts of N (4 ng/ml for our ELISA, 1 ng/ml for the dipstick test). In total, 18 samples diluted 1/3 were tested and a correlation was observed for 16 out of 18 samples between both techniques, validating our sandwich ELISA. Some PCR negative samples #6 and #79 were found positive while PCR positive samples #14, #22, and #30 were found negative with both techniques. These discrepancies will need to be further analyzed. However, samples #14, #22, and #30 are from patients in the early phase of infection (3, 8, and 2 days postinfection, respectively) suggesting that the concentration of N may be low. On the other hand, the ELISA is able to detect N in the sample #79 39 days postinfection, suggesting that the presence of N might persist even after recovery from infection. The development of a reliable test based on VHHs will be performed in the future by using a large number of human samples. This test can be adapted to an ultrasensitivity Simoa assay that could increase the sensitivity 3000-fold compared with that of the commercially available N protein ELISA kit assay (48). The ultrasensitivity of Simoa assays should provide a quantitative resolution of N concentrations and enable to detect N even at the earliest stages of infection. An alternative could be the use of a highly sensitive Luciferase-Linked Immunosorbent Assay (LuLISA) relying on anti N VHHs expressed in tandem with the catalytic domain of the enzyme luciferase (nanoKAZ) (49).

## Experimental procedures

### Production of recombinant N from SARS-CoV-2

An optimized synthetic gene (GenBank MN908947) was cloned in the pETM11 expression vector allowing the production of N fused to an N-terminal (His)_6_ tag. Production and purification of N have been described by Grzelak *et al.* (40).

### Quality assessment of N

Prior to further experiments, both VHHs and N underwent a series of tests to assess their integrity, solubility, and stability. The tests are adapted from previously published approach (50) and the ARBRE-MOBIEU P4EU recommendations (https://arbre-mobieu.eu/guidelines-on-protein-quality-control/).

#### UV spectroscopy quantification

Protein quantification at 280 nm was carried out by recording a full spectrum between 240 and 340 nm. Detection of nucleotides at 260 nm and scattering at 340 nm were also checked. Measurements were done at room temperature in a 1 cm quartz cell, reference 105.202-QS.10 (Hellma), using a JASCO V-650 spectrophotometer (JASCO Corporation). A baseline subtraction at 340 nm was performed with the Spekwin32 software (F. Menges “Spekwin32 – optical spectroscopy software,” Version 1.72.2, 2016, http://www.effemm2.de/spekwin/) to accurately calculate the protein concentration.

#### Dynamic light scattering (DLS) experiments

DLS was performed on a DynaPro Plate Reader III (Wyatt) to confirm that the samples did not contain aggregates. Experiments were performed in triplicate in a 384-well microplate (Corning ref 3540), with 20 acquisitions of 10 s each, monitored with the DYNAMICS version V7.9.1.3 software (Wyatt). The N protein stored at 4 °C was monitored for 3 weeks, and an overnight experiment at 37 °C was also performed. The VHHs were monitored at 20 °C just after their purification.

#### Sodium dodecyl sulfate–polyacrylamide gel electrophoresis (SDS-PAGE)

Polyacrylamide gel electrophoresis (PAGE) was performed using NuPAGE Novex 4–12% Bis-Tris gel (Invitrogen) according to the manufacturer's instructions. PageRuler Prestained Protein ladder was used as molecular weight maker and Instant Blue (Expedeon) was used to stain the SDS-PAGE gel.

#### Intact mass measurement by LC-ESI-MS

Recombinant N was diluted to 0.2 μM in 0.15 % formic acid (pH 2.5). 50 μl (10 pmol) was loaded onto an ACQUITY UPLC BEH C4 Trap column (2.1 μm × 5 mm, Waters Corporation), and desalted for 2 min at 100 μl/min with 0.15 % formic acid, pH 2.5. The protein was eluted into the mass spectrometer with a quick linear gradient of acetonitrile from 5 to 90 % in 2 min, at 60 μl/min. Mass spectra were acquired in resolution and positive ion-mode (*m/z* 400–2000) on a Synapt G2-Si HDMS mass spectrometer (Waters Corporation). To ensure mass accuracy, a Glu-1-Fibrinopeptide B solution (100 fmol/μl in 50% acetonitrile, 0.1% formic acid) was continuously infused through the reference probe of the electrospray source.

#### Sedimentation velocity analysis

Sedimentation velocity experiments were carried out at 42,000 rpm and 20 °C in an Optima analytical ultracentrifuge, using 12-mm aluminum-Epon double-sector centerpieces in an 55Ti rotor. Protein concentrations were recorded in continuous mode using absorbance at 230, 271 nm. N proteins were studied at 0.15 mg/ml. The partial specific volume, solvent density, and viscosity, calculated with SEDNTERP (http://www.jphilo.mailway.com/download.htm), were 0.724 ml/g, 1.012 g/cm^3^, and 0.01045 P, respectively. The data recorded from moving boundaries were analyzed in terms of continuous size distribution function of sedimentation coefficient C(S) using the program SEDFIT (51).

### Production of the N-terminal domain (NTD) of N

The gene encoding residues 1–200 of N (GenBank: YP_009724397.2) were retrieved by polymerase chain reaction (PCR). The amplicon was subcloned into a pET23-derived plasmid encoding an His6 tag at 3′end. Sequencing verified that no mutations were introduced during the process. The recombinant protein was expressed in *E.coli* SHuffle C3029H cells (New England Biolabs) and purified from a soluble cytoplasmic extract, as described above for the whole N. About 15 mg of purified protein was systematically obtained from 1 L of culture medium.

### Production of native nucleoprotein: cell extracts and virus inactivation

Vero-NK (African Green Monkey Kidney) cells were infected with the SARS-CoV-2 virus (BetaCoV/France/IDF0372/2020) at a MOI of 10^−2^. An uninfected control was also produced in the same conditions. After 24 h of incubation, the cells were washed with 150 mM NaCl and 50 mM Tris HCl pH7.5 (TBS), and the cell monolayer was scratched. The cells were centrifuged and the pellet was resuspended in TBS-2% Triton X100 and incubated at 37 °C for 15 min before being sonicated. Cells were centrifuged and β-propiolactone (1/50) was added to the supernatant before being incubated for 24 h at 4 °C, then 24 h at 20 °C. The virus inactivation was controlled before the use of cell extracts for ELISA.

The SARS-CoV-2 virus was also inactivated with β-propiolactone (1/50) in TBS for 24 h at 4 °C, then 24 h at 20 °C. The virus inactivation was then controlled. To permeabilize the viral membrane, the virus was incubated for 15 min at 37 °C in PBS-2% Triton X100.

### Alpaca immunization

Animal procedures were performed according to the French legislation and in compliance with the European Communities Council Directives (2010/63/UE, French Law 2013-118, February 6, 2013). The Animal Experimentation Ethics Committee of Pasteur Institute (CETEA 89) approved this study (2020-27412). One young adult male alpaca (*Lama pacos*) was immunized at days 0, 17, and 24 with 150 μg of recombinant N. The immunogen was mixed with Freund complete adjuvant for the first immunization and with Freund incomplete adjuvant for the following immunizations. The immune response was monitored by titration of serum samples by ELISA on coated N. The bound alpaca antibodies were detected with polyclonal rabbit anti-alpaca IgGs (52).

### Library construction and phage display

The blood of the immunized animal (about 300 ml) was collected and the peripheral blood lymphocytes were isolated by centrifugation on a Ficoll (Cytiva, Velizy, France) discontinuous gradient and stored at −80 °C until further use. Total RNA and cDNA were obtained as previously described (52). A nested PCR was performed with IgG-specific primers designed in our lab. In the first step, five sets of PCR primers were used to amplify the VH-CH1-CH2 and VHH-CH2 fragments. The bands corresponding to the VHH-CH2 regions were purified on an agarose gel. Next, VHH regions were specifically reamplified with three sets of VHH-specific PCR primers complementary to the 5′ and 3′ ends of the amplified product and incorporating Sfi1 and Not1 restriction sites at the ends of the VHH genes. The PCR products were digested and ligated into a pHEN6 phagemid vector.

Phage display technology allows the selection of antigen specific phage-VHHs. A large number of phage-VHHs (10^13^) were used to perform three rounds of panning. A different blocking agent was used for each round, respectively 2% skimmed milk, Licor blocking buffer (Biosciences) diluted into PBS at a 1:4 ratio and 4% BSA. After the blocking step, phage-VHHs were incubated with antigen precoated immunotubes for 2 h on a wheel at room temperature. To remove nonspecific binders, a 6× PBS Tween 0.1% and 4× PBS washing procedure was performed, specific phage-VHHs were then eluted in 100 mM triethylamine (TEA) for 5 min on a wheel, and the excess TEA was neutralized immediately with 1 M Tris-HCl, pH 7.6. *E. coli* TG1 at exponential growth phase were then infected with eluted phage-VHHs and incubated at 37 °C for 30 min without stirring and 30 min under stirring. The remaining bacteria were centrifuged at 4000 rpm for 15 min, and the pellet was resuspended in 1 ml of 2YT, which was spread on a 2YT+ampicillin (A) Bio-assay dish (24 cm × 24 cm) and incubated overnight at 30 °C. Bacteria were recovered the next day with 4 ml of 2YT containing 8% of DMSO (Dimethyl sulfoxide Sigma-Aldrich) and were stored at −80 °C in aliquots of 1 ml.

### Selection of specific phage-VHHs and ELISA

Individual colonies from the second and the third round of panning were picked from Petri dishes and were cultured in a 96-well plate (Plate I) (Cell star, Greiner Bio-one) containing 200 μl of 2YT +A/well overnight at 37 °C with shaking. This plate (Plate I) was used to seed a secondary plate (Plate II) to express phage-VHH. Three microliters of each colony were cultured in 200 μl of 2YT A in a 96-deepwell plate (MasterBlock, Greiner Bio-one) (Plate II). After a 1 h 30 min incubation at 37 °C with shaking, the bacteria present in each well were infected with 1 × 10^9^ VCS M13 helper phages. The plate II was then incubated for 30 min at 37 °C without shaking, followed by 30 min at 37 °C with shaking and then centrifuged at 2500 rpm for 10 min. The pelleted cells in each well were resuspended in 500 μl of 2YT+A+Kanamycin (K)+IPTG. The cultures were then incubated overnight at 30 °C with shaking to allow expression of phage-VHHs by bacteria. Each well contained a single selected phage-VHH. In parallel, plate III (Nunc Thermo Scientific) was coated with antigen overnight at 4 °C.

The following day, plates III were first saturated with PGT (PBS-Gelatin 0.5%-Tween 0.1%) for 30 min at 37 °C (100 μl/well). Plates II were centrifuged at 2500 rpm for 10 min to precipitate bacteria and to retrieve the supernatant containing phage-VHHs. Phage-VHHs were then diluted with PGT in a ratio of 1/5, transferred into plates III, and incubated at 37 °C for 1 h. Between each ELISA step, plates were washed six times with PBS-Tween 0.1%. Anti-phage M13 IgG conjugated to HRP (Sinobiologicals #11973-MM05T-H, lot number H014N02001) diluted in PGT (100 μl) at 1/5000 was added for 1 h at 37 °C. Subsequently, the reactions were developed by adding 100 μl of OPD (o-Phenylenediamine, Dako) and stopped by adding 50 μl of 3 M HCl. The optical density was measured spectrophotometrically at 490 nm using a Magellan microplate reader (Sunrise Tecan). A clone was considered positive when the SNR (signal-to-noise ratio) was greater than or equal to 10.

### Expression of VHHs

The pHEN6 vector encodes a His tag and a c-myc tag that allows to express free VHHs in the periplasm and to purify them. Transformed *E. coli* TG1 cells expressed VHH in the periplasm after overnight induction with 0.25 mM IPTG at 16 °C.

The coding sequences of the selected VHHs in the vector pHEN6 were subcloned into a bacterial expression vector pASK (IBA) encoding a C terminal strep tag using NcoI and NotI restriction sites. Transformed *E. coli* XL1 cells expressed VHH in the periplasm after overnight induction with anhydrotetracycline (200 μg/l) at 30 °C. Purified VHHs were isolated on Strep-Tactin affinity columns from periplasmic extracts treated by 10 U/ml Benzonase Nuclease (Merck) and Complete protease inhibitor (Roche), according to the manufacturer's instructions, followed by size exclusion chromatography with a Superdex 75 column (Cytiva). The VHHs were assessed for quality using the protocol described above for N.

### Biotinylation of VHHs

The VHHs were biotinylated using the EZ-linkSulfo-NHS-biotin kit (Thermo) according to manufacturer's instructions.

### Enzyme-linked immunosorbent assay (ELISA)

A modified version of a standard ELISA was used to test for the presence of VHH. Maxisorp Nunc-Immuno plates (Thermo Scientific) were coated with 1 μg/ml of recombinant protein or cell extracts (1/1000) overnight at 4 °C. Plates were washed with PBS containing 0.1% Tween 20. Strep-tagged VHHs were diluted in PBS containing 1% BSA and 0.1% Tween 20. After 1 h incubation at 37 °C, plates were washed again before adding an anti-strep-tag mouse antibody (the monoclonal antibody C23-21 has been obtained at the Antibody Engineering platform and is now commercialized by MiliporeSigma #MAC143) followed by a peroxidase-labeled goat anti-mouse immunoglobulins (Vector #PI-2000 lot number 2C1212). TMB (3,3′-5′5-tetramethylbenzidine, SeraCare) was used as substrate. The optical density was measured spectrophotometrically at 450 nm using a Magellan microplate reader (Sunrise Tecan).

The recombinant proteins used for the coating were: SARS-CoV-1 and SARS-CoV-2 N produced in *E.coli* as described above, seasonal human coronaviruses N from Sino Biological, and SARS-CoV-2 Spike protein (40).

### Nasopharyngeal samples

Patients were sampled for nasopharyngeal swabs after a median duration of 9 days (interquartile range, 2–39) after disease onset. Nasopharynx specimens were obtained with sterile dry swabs (COPAN LQ Stuart Transport Swab, COPAN Italia SpA), which were rotated five times around the inside of each nostril while applying constant pressure. Nasopharynx swabs were collected in the office under strict aseptic conditions. Prior to ELISA analysis, nasopharyngeal swabs (1 ml) were treated in a P3 laboratory for viral decontamination. Briefly, samples were treated with Triton X100 (TX100) 1% (v/v) for 2 h at room temperature. Nasopharyngeal viral loads were determined using RdRp-IP4 quantitative RT-PCR designed at the Institut Pasteur (National Reference Center for Respiratory Viruses) to target a section of the RdRp gene based on the first sequences of SARS-CoV-2 made available on the Global Initiative on Sharing All Influenza Data database on Jan 11, 2020 (53). Primer and probe sequences: nCoV_IP4-14059Fw GGTAACTGGTATGATTT CG; nCoV_IP4-14146Rv CTGGTCAAGGTTAATATAGG; nC oV_IP4-14084Probe(+) TCATACAAACCACGCCAGG [5′]Fam [3′]BHQ-1. The work described was carried out in accordance with the Code of Ethics of the World Medical Association (Declaration of Helsinki) for experiments involving humans.

### Detection of nucleoprotein in solution by sandwich ELISA

VHH NTD-E4 or NTD-B6 was coated on Maxisorp Nunc-Immuno plates at 1 μg/ml. After washing with 0.1% Tween 20 in PBS, recombinant N, permeabilized virus diluted at different concentrations or nasal swabs were added for 1 h at 37 °C. Then biotinylated VHHs (2 μg/ml) were added for 1 h at 37 °C followed by the addition of a peroxidase labeled streptavidin (Jackson ImmunoResearch #016-030-084, lot number 147172).

### Detection of nucleoprotein in solution by an immunochromatographic assay

The Rapid SARS-CoV-2 Antigen Test Card (MP biomedicals) was used for the detection of N according to manufacturer's instructions.

### Epitope mapping by hydrogen deuterium exchange–mass spectrometry

A summary of the main HDX-MS experimental conditions is provided in Table S1 (54). The quality and purity of N were assessed by intact mass analysis (Fig. 1). All labeling was performed at room temperature in deuterated PBS 1X buffer, pD 7.4 (labeling buffer), unless specified.

#### Sample preparation

N was labeled in the presence and absence of each VHH. Each complex was formed by mixing 6.6 μl of N (12.7 μM in PBS 1X, monomer concentration) with 1.4 μl of VHH (stock solution at ∼ 70 μM in PBS 1X). A control sample (Apo-state) was prepared in parallel by replacing the VHH solution with PBS 1X. After 30 min incubation at room temperature, the labeling was initiated by adding 62 μl of labeled buffer (final D_2_0/H_2_0 ratio = 88.6/11.4%). The final concentration of each protein (N = 1.2 μM; VHH = ∼1.4 μM) was carefully selected to avoid the dissociation of the homodimer (55) and to ensure than >95% of N remained in complex during the exchange reaction. Continuous labeling was performed for t = 0.16, 1, 5, 10, 30, 60, and 120 min. Aliquots of 10 μl (*i.e.*, 12 pmol of N with or without 14 pmol of VHH) were removed and quenched upon mixing with 50 μl of an ice-cold solution of 2% formic acid, 3 M urea to decrease the pH to 2.50 (final D_2_0/H_2_0 ratio = 14.8/85.2%). Quenched samples were immediately snap frozen in liquid nitrogen and stored at −80 °C.

Undeuterated samples were obtained following the same experimental procedure. A fully deuterated control was prepared by mixing 6.6 μl of N (12.7 μM, monomer concentration) with 1.4 μl of PBS 1X and 62 μl of labeled buffer supplemented with 8 M urea-d4 (final D_2_0/H_2_0 ratio = 88.6/11.4%). After 21 h incubation at room temperature, samples were quenched as described above using a cold solution of 2% formic acid, 1.6 M urea. All samples were prepared in triplicate for each time point and condition (with the exception of the 120 min time point for both VHH E10 and H3 where two replicates were acquired).

#### LC-MS data acquisition

Quenched samples were thawed and immediately injected onto an HDX manager connected to two nanoACQUITY UPLC M-Class pumps (Waters Corporation). The temperature of the HDX manager was maintained at 0 °C to minimize back exchange. Fifty microliters of each labeled sample (*i.e.*, 10 pmol of N with or without 11.7 pmol of VHH) was digested using an in-house packed column (2.0 × 20 mm, 63 μl bed volume) of immobilized pig pepsin agarose beads (Thermo Scientific) for 2 min at 20 °C. Peptides were directly trapped and desalted onto a C18 Trap column (VanGuard BEH 1.7 μm, 2.1 × 5 mm, Waters Corporation) at a flow rate of 100 μl/min (0.15% formic acid) and separated by an 8 min linear gradient of 5–30% acetonitrile followed by a short 2 min increase from 30% to 40% of acetonitrile at 40 μl/min using an ACQUITY UPLC BEH C18 analytical column (1.7 μm, 1 × 100 mm, Waters Corporation, Milford, MA). After each run, the pepsin column was manually cleaned with two consecutive injections of 1% formic acid, 5% acetonitrile, 1.5 M guanidinium chloride, pH 1.7. Blank injections were performed between each run to confirm the absence of carryover.

Mass spectra were acquired in resolution and positive ion-mode (*m/z* 50–2000) on a Synapt G2-Si HDMS mass spectrometer (Waters Corporation) equipped with a standard ESI source and lock-mass correction. Peptic peptides were identified in undeuterated samples by a combination of data-independent acquisition (MS^E^) and exact mass measurement (below 10 ppm mass error) using the same chromatographic conditions than for the deuterated samples. Four distinct MS^E^ trap collision energy ramps were employed to optimize the efficiency of the fragmentation: 10–30 V (low), 15–35 V (medium), 20–45 V (high), and 10–45 V (mixed mode).

#### Data processing

The initial N peptide map was generated by database searching in ProteinLynX Global server 3.0 (Waters Corporation) using the following processing and workflow parameters: low and elevated intensity thresholds set to 100.0 and 50.0 counts; intensity threshold sets to 750.0 counts; automatic peptide and fragment tolerance; nonspecific primary digest reagent; false discovery rate sets to 4%. Each fragmentation spectrum was manually inspected for assignment confirmation. The N-arm (residues 1–45), LCK (residues 180–246), CTD N-terminal region (residues 247–267), and the C-tail (residues 363–419) domains of N contain a high proportion of residues not tolerated by pig pepsin (*i.e.*, Proline, Lysine, Histidine, or Arginine) resulting in a lack of sequence coverage or resolution. Type XIII protease from *Aspergillus saitoi* (Sigma Aldrich) either immobilized on POROS 20-AL beads (Applied Biosystems, Bedford, MA) or in solution did not improve the final sequence coverage and resolution. Pig pepsin was therefore selected to perform local HDX analysis. The peptide map was further refined in DynamX 3.0 (Waters Corporation) using the following Import PLGS results filters: minimum intensity = 3000; minimum products per amino acid = 0.15; minimum score = 6.5; maximum MH+ error (ppm) = 10; file threshold = 2.

DynamX 3.0 was used to extract the centroid masses of all peptides selected for HDX-MS. One unique charge state was used per peptide and no back-exchange correction was performed. HDX-MS results are reported as relative deuterium exchange level expressed in either mass unit or fractional exchange. Fractional exchange data were calculated by dividing the experimental uptake value by the theoretically maximum number of exchangeable backbone amide hydrogens that could be replaced into each peptide in 88.6% excess deuterium. Overlapping peptides covering the same region were only used to increase the spatial resolution if their experimental back-exchange values were similar (difference <10%, Table S1). The MEMHDX software (56) was used to visualize and statistically validate HDX-MS datasets (Wald test, false discovery rate of 1%, biological threshold sets to 3%, Table S1).

### Kinetic characterization by surface plasmon resonance (SPR)

Experiments were performed using a Biacore T200 instrument (GE Healthcare) equilibrated at 25 °C in SPR buffer (PBS-300 mM NaCl containing 0.1% Tween-20, 0.2 mg/ml BSA and 100 μM EDTA).

Approximately 500 RU (1RU ≈ 1 pg·mm^−2^) of N were captured noncovalently on an NiCl_2_-loaded NTA sensor chip (GE Healthcare). VHHs were then injected at 30 μl/min for 300 s (E10-3, D12-3, NTD B6-1 and NTD E4-3) or 700 s (E7-2, G9-1 and H3-3) to monitor the association of the VHH-N complexes, after which SPR buffer was injected for another 300 s or 1200 s to monitor the dissociation of the complexes.

Finally, the surface of the sensor chip was regenerated by injecting sequentially EDTA 0.5 M and SDS 0.1% for 60 s.

Association and dissociation profiles were analyzed with the BiacoreT200 evaluation software, assuming a 1:1 interaction, which allowed to determine the association (k_on_) and dissociation (k_off_) rates of the interactions, as well as their equilibrium constants (K_d_).

### Immunofluorescence assays

FRhK4 (Fetal Rhesus monkey Kidney) cells were grown in 96-well plate coated with poly-D-lysine. Infection was performed at 37 °C on exponentially growing cells at a multiplicity of infection of 10^2^ in order to have approximately one out of two cells infected with SARS-CoV-2 virus after 24 h. Cells were fixed 20 min at 4 °C with PBS containing 2% PFA (v/v) and permeabilized 10 min at 4 °C with 0.2% Triton X100 in PBS (v/v). Cells were incubated at room temperature with PBS-BSA 3% 1 h without VHH then 1 h with biotinylated VHH at 1 μg/ml or rabbit polyclonal antibodies against N- SARS-CoV-2 as a control of cell infection. To label the VHH a streptavidin Alexa Fluor 488 (Thermo Fisher) in PBS-BSA 3% was incubated for 1 h at room temperature according to manufacturer's instructions. Immunofluorescence was observed at 40× on a fluorescent microscope (Zeiss).

### SARS-CoV-2 intranasal inoculation and tissue imaging

Animal procedures were performed according to the French legislation and in compliance with the European Communities Council Directives (2010/63/UE, French Law 2013-118, February 6, 2013). The Animal Experimentation Local Ethics Committee (CETEA 89) approved this study (2020-0023 and dap200008). The animals were housed and manipulated in isolators in a Biosafety level-3 facility. Male *Mesocricetus auratus* Syrian hamsters of 5–6 weeks of age (Janvier, Le Genest Saint Isle, France) were intranasally inoculated under anesthesia (intraperitoneal injection of ketamine (200 mg/kg) and xylazine (10 mg/kg)) with 100 μl of SARS-CoV-2 (50 μl/nostril) (isolate IDF0372/2020, EVAg collection, Ref-SKU: 014V-03890) at 6 × 10^4^ PFU (plaque-forming units))- or physiological solution as previously described (41).

Lungs were collected at 4 days postinfection, formalin-fixed after transcardial perfusion of hamsters with a physiological solution containing heparin (5 × 10^3^ U/ml, Sanofi) followed by 4% paraformaldehyde in phosphate buffer. Tissues were postfixed by incubation in the same fixative during 1 week, cryoprotected by incubation in 30% sucrose in PBS overnight, and then embedded in Tissue-tek (Sakura). Lung 20-μm-thick transverse sections were obtained using a cryostat (CM3050S, Leica) and were thaw-mounted onto coated glass slides (Superfrost Plus). Eight-week-old female C57BL/6JRj (C57BL/6) mice (Janvier Labs, Le Genest St Isle, France) were anesthetized and inoculated intranasally with 6 × 10^4^ PFU of SARS-CoV-2 B.1.351 and P.1 variants as previously described (55). Lungs were collected at 3 days postinfection and fixed by submersion in 10% phosphate buffered formalin for 7 days. Paraffin-embedded 4 μm-thick sections were used for immunostaining.

Antigen retrieval was performed by incubating sections for 20 min in citrate buffer 0.1 M pH 6.0 at 96 °C and then blocked in 0.4% Triton, 4% fetal bovine serum (Sigma), and 10 % goat serum (ThermoScientific). They were incubated overnight at 4 °C with biotinylated VHHs diluted 1/500, rinsed in PBS and followed by a 2 h-incubation step with Alexa 568-conjugated streptavidin (Jackson ImmunoResearch Laboratories) at room temperature. Fluorescent sections were stained with the nuclear dye HOESCHT and then mounted in Fluoromount solution (Invitrogen).

## Data availability

The datasets generated during and/or analyzed during the current study are available from the corresponding author on reasonable request.

## Ethics approval statement for animal studies

Animal procedures were performed according to the French legislation and in compliance with the European Communities Council Directives (2010/63/UE, French Law 2013-118, February 6, 2013). The Animal Experimentation Ethics Committee of Pasteur Institute (CETEA 89) approved the alpaca study (2020-27412) and the Syrian hamster study (2020-0023).

## Supporting information

This article contains supporting information (Table S1 and Figs. S1–S9).

## Conflict of interest

The authors declare that they have no conflicts of interest with the contents of this article.

## References

[1] Su S., Wong G., Shi W., Liu J., Lai A.C.K., Zhou J., Liu W., Bi Y., Gao G.F. (2016). Epidemiology, genetic recombination, and pathogenesis of coronaviruses. Trends Microbiol..

[2] De Wit E., Van Doremalen N., Falzarano D., Munster V.J. (2016). SARS and MERS: Recent insights into emerging coronaviruses. Nat. Rev. Microbiol..

[3] Wu D., Wu T., Liu Q., Yang Z. (2020). The SARS-CoV-2 outbreak: What we know. Int. J. Infect. Dis..

[4] McBride R., van Zyl M., Fielding B.C. (2014). The coronavirus nucleocapsid is a multifunctional protein. Viruses.

[5] Takeda M., Chang C.K., Ikeya T., Güntert P., Chang Y.H., Hsu Y.L., Huang T.H., Kainosho M. (2008). Solution structure of the C-terminal dimerization domain of SARS coronavirus nucleocapsid protein solved by the SAIL-NMR method. J. Mol. Biol..

[6] Surjit M., Liu B., Chow V.T., Lal S.K. (2006). The nucleocapsid protein of severe acute respiratory syndrome-coronavirus inhibits the activity of cyclin-cyclin-dependent kinase complex and blocks S phase progression in mammalian cells. J. Biol. Chem..

[7] Saikatendu K.S., Joseph J.S., Subramanian V., Neuman B.W., Buchmeier M.J., Stevens R.C., Kuhn P. (2007). Ribonucleocapsid formation of severe acute respiratory syndrome coronavirus through molecular action of the N-terminal domain of N protein. J. Virol..

[8] Chen C.Y., Chang C.K., Chang Y.W., Sue S.C., Bai H.I., Riang L., Hsiao C.D., Huang T.H. (2007). Structure of the SARS coronavirus nucleocapsid protein RNA-binding dimerization domain suggests a mechanism for helical packaging of viral RNA. J. Mol. Biol..

[9] Zeng W., Liu G., Ma H., Zhao D., Yang Y., Liu M., Mohammed A., Zhao C., Yang Y., Xie J., Ding C., Ma X., Weng J., Gao Y., He H., Jin T. (2020). Biochemical characterization of SARS-CoV-2 nucleocapsid protein. Biochem. Biophys. Res. Commun..

[10] Papageorgiou N., Lichière J., Baklouti A., Ferron F., Sévajol M., Canard B., Coutard B. (2016). Structural characterization of the N-terminal part of the MERS-CoV nucleocapsid by X-ray diffraction and small-angle X-ray scattering. Acta Crystallogr D Struct. Biol.

[11] Nguyen V.D., Hatahet F., Salo K.E., Enlund E., Zhang C., Ruddock L.W. (2011). Pre-expression of a sulfhydryl oxidase significantly increases the yields of eukaryotic disulfide bond containing proteins expressed in the cytoplasm of E.coli. Microb. Cell Fact..

[12] Peng Y., Du N., Lei Y., Dorje S., Qi J., Luo T., Gao G.F., Song H. (2020). Structures of the SARS-CoV-2 nucleocapsid and their perspectives for drug design. EMBO J..

[13] Dinesh D.C., Chalupska D., Silhan J., Koutna E., Nencka R., Veverka V., Boura E. (2020). Structural basis of RNA recognition by the SARS-CoV-2 nucleocapsid phosphoprotein. PLoS Pathog..

[14] Liu L., Liu W., Zheng Y., Jiang X., Kou G., Ding J., Wang Q., Huang Q., Ding Y., Ni W., Wu W., Tang S., Tan L., Hu Z., Xu W. (2020). A preliminary study on serological assay for severe acute respiratory syndrome coronavirus 2 (SARS-CoV-2) in 238 admitted hospital patients. Microbes Infect..

[15] Hamers-Casterman C., Atarhouch T., Muyldermans S., Robinson G., Hamers C., Songa E.B., Bendahman N., Hamers R. (1993). Naturally occurring antibodies devoid of light chains. Nature.

[16] Van Audenhove I., Boucherie C., Pieters L., Zwaenepoel O., Vanloo B., Martens E., Verbrugge C., Hassanzadeh-Ghassabeh G., Vandekerckhove J., Cornelissen M., De Ganck A., Gettemans J. (2014). Stratifying fascin and cortactin function in invadopodium formation using inhibitory nanobodies and targeted subcellular delocalization. FASEB J..

[17] Traenkle B., Rothbauer U. (2017). Under the microscope: Single-domain antibodies for live-cell imaging and super-resolution microscopy. Front. Immunol..

[18] Perruchini C., Pecorari F., Bourgeois J.P., Duyckaerts C., Rougeon F., Lafaye P. (2009). Llama VHH antibody fragments against GFAP: Better diffusion in fixed tissues than classical monoclonal antibodies. Acta Neuropathol..

[19] Li T., Bourgeois J.P., Celli S., Glacial F., Le Sourd A.M., Mecheri S., Weksler B., Romero I., Couraud P.O., Rougeon F., Lafaye P. (2012). Cell-penetrating anti-GFAP VHH and corresponding fluorescent fusion protein VHH-GFP spontaneously cross the blood-brain barrier and specifically recognize astrocytes: Application to brain imaging. FASEB J..

[20] Li T., Vandesquille M., Koukouli F., Dudeffant C., Youssef I., Lenormand P., Ganneau C., Maskos U., Czech C., Grueninger F., Duyckaerts C., Dhenain M., Bay S., Delatour B., Lafaye P. (2016). Camelid single-domain antibodies: A versatile tool for in vivo imaging of extracellular and intracellular brain targets. J. Control Release.

[21] Newnham L.E., Wright M.J., Holdsworth G., Kostarelos K., Robinson M.K., Rabbitts T.H., Lawson A.D. (2015). Functional inhibition of β-catenin-mediated Wnt signaling by intracellular VHH antibodies. MAbs.

[22] Vanlandschoot P., Stortelers C., Beirnaert E., Ibañez L.I., Schepens B., Depla E., Saelens X. (2011). Nanobodies®: New ammunition to battle viruses. Antiviral Res.

[23] Lafaye P., Li T. (2018). Use of camel single-domain antibodies for the diagnosis and treatment of zoonotic diseases. Comp. Immunol. Microbiol. Infect. Dis..

[24] Forsman A., Beirnaert E., Aasa-Chapman M.M., Hoorelbeke B., Hijazi K., Koh W., Tack V., Szynol A., Kelly C., McKnight A., Verrips T., de Haard H., Weiss R.A. (2008). Llama antibody fragments with cross-subtype human immunodeficiency virus type 1 (HIV-1)-neutralizing properties and high affinity for HIV-1 gp120. J. Virol..

[25] McCoy L.E., Quigley A.F., Strokappe N.M., Bulmer-Thomas B., Seaman M.S., Mortier D., Rutten L., Chander N., Edwards C.J., Ketteler R., Davis D., Verrips T., Weiss R.A. (2012). Potent and broad neutralization of HIV-1 by a llama antibody elicited by immunization. J. Exp. Med..

[26] Hultberg A., Temperton N.J., Rosseels V., Koenders M., Gonzalez-Pajuelo M., Schepens B., Ibañez L.I., Vanlandschoot P., Schillemans J., Saunders M., Weiss R.A., Saelens X., Melero J.A., Verrips C.T., Van Gucht S. (2011). Llama-derived single domain antibodies to build multivalent, superpotent and broadened neutralizing anti-viral molecules. PLoS One.

[27] Ashour J., Schmidt F.I., Hanke L., Cragnolini J., Cavallari M., Altenburg A., Brewer R., Ingram J., Shoemaker C., Ploegh H.L. (2015). Intracellular expression of camelid single-domain antibodies specific for influenza virus nucleoprotein uncovers distinct features of its nuclear localization. J. Virol..

[28] Laursen N.S., Friesen R.H.E., Zhu X., Jongeneelen M., Blokland S., Vermond J. (2018). Universal protection against influenza infection by a multidomain antibody to influenza hemagglutinin. Science.

[29] Thys B., Schotte L., Muyldermans S., Wernery U., Hassanzadeh-Ghassabeh G., Rombaut B. (2010). *In vitro* antiviral activity of single domain antibody fragments against poliovirus. Antivir. Res.

[30] Harmsen M.M., De Haard H.J. (2007). Properties, production, and applications of camelid single-domain antibody fragments. Appl. Microbiol. Biotechnol..

[31] van der Vaart J.M., Pant N., Wolvers D., Bezemer S., Hermans P.W., Bellamy K., Sarker S.A., van der Logt C.P., Svensson L., Verrips C.T., Hammarstrom L., van Klinken B.J. (2006). Reduction in morbidity of rotavirus induced diarrhoea in mice by yeast produced monovalent llama-derived antibody fragments. Vaccine.

[32] Tarr A.W., Lafaye P., Meredith L., Damier-Piolle L., Urbanowicz R.A., Meola A., Jestin J.L., Brown R.J., McKeating J.A., Rey F.A., Ball J.K., Krey T. (2013). An alpaca nanobody inhibits hepatitis C virus entry and cell-to-cell transmission. Hepatology.

[33] Wrapp D., De Vlieger D., Corbett K.S., Torres G.M., Wang N., Van Breedam W. (2020). Structural basis for potent neutralization of Betacoronaviruses by single-domain camelid antibodies. Cell.

[34] Huo J., Le Bas A., Ruza R.R., Duyvesteyn H.M.E., Mikolajek H., Malinauskas T., Tan T.K., Rijal P., Dumoux M., Ward P.N., Ren J., Zhou D., Harrison P.J., Weckener M., Clare D.K. (2020). Neutralizing nanobodies bind SARS-CoV-2 spike RBD and block interaction with ACE2. Nat. Struct. Mol. Biol..

[35] Esparza T.J., Martin N.P., Anderson G.P., Goldman E.R., Brody D.L. (2020). High affinity nanobodies block SARS-CoV-2 spike receptor binding domain interaction with human angiotensin converting enzyme. Sci. Rep..

[36] Xiang Y., Nambulli S., Xiao Z., Liu H., Sang Z., Duprex W.P., Schneidman-Duhovny D., Zhang C., Shi Y. (2020). Versatile and multivalent nanobodies efficiently neutralize SARS-CoV-2. Science.

[37] Chi X., Liu X., Wang C., Zhang X., Li X., Hou J., Ren L., Jin Q., Wang J., Yang W. (2020). Humanized single domain antibodies neutralize SARS-CoV-2 by targeting the spike receptor binding domain. Nat. Commun..

[38] Anderson G.P., Liu J.L., Esparza T.J., Voelker B.T., Hofmann E.R., Goldman E.R. (2021). Single-domain antibodies for the detection of SARS-CoV-2 nucleocapsid protein. Anal. Chem..

[39] Koenig P.A., Das H., Liu H., Kümmerer B.M., Gohr F.N., Jenster L.M., Schiffelers L.D.J., Tesfamariam Y.M., Uchima M., Wuerth J.D., Gatterdam K., Ruetalo N., Christensen M.H., Fandrey C.I., Normann S. (2021). Structure-guided multivalent nanobodies block SARS-CoV-2 infection and suppress mutational escape. Science.

[40] Grzelak L., Temmam S., Planchais C., Demeret C., Tondeur L., Huon C., Guivel-Benhassine F., Staropoli I., Chazal M., Dufloo J., Planas D., Buchrieser J., Rajah M.M., Robinot R., Porrot F. (2020). A comparison of four serological assays for detecting anti–SARS-CoV-2 antibodies in human serum samples from different populations. Sci. Transl. Med..

[41] de Melo G.D., Lazarini F., Levallois S., Hautefort C., Michel V., Larrous F., Verillaud B., Aparicio C., Wagner S., Gheusi G., Kergoat L., Kornobis E., Donati F., Cokelaer T., Hervochon R. (2021). COVID-19-related anosmia is associated with viral persistence and inflammation in human olfactory epithelium and brain infection in hamsters. Sci. Transl. Med..

[42] Hou Y.J., Okuda K., Edwards C.E., Martinez D.R., Asakura T., Dinnon K.H., Kato T., Lee R.E., Yount B.L., Mascenik T.M., Chen G., Olivier K.N., Ghio A., Tse L.V., Leist S.R. (2020). SARS-CoV-2 reverse genetics reveals a variable infection gradient in the respiratory tract. Cell.

[43] Sungnak W., Huang N., Bécavin C., Berg M., Queen R., Litvinukova M., Talavera-López C., Maatz H., Reichart D., Sampaziotis F., Worlock K.B., Yoshida M., Barnes J.L. (2020). SARS-CoV-2 entry factors are highly expressed in nasal epithelial cells together with innate immune genes. Nat. Med..

[44] Leung K., Shum M.H., Leung G.M., Lam T.T., Wu J.T. (2021). Early transmissibility assessment of the N501Y mutant strains of SARS-CoV-2 in the United Kingdom, October to November 2020. Euro Surveill..

[45] Tegally H., Wilkinson E., Lessells R.J., Giandhari J., Pillay S., Msomi N., Mlisana K., Bhiman J.N., von Gottberg A., Walaza S., Fonseca V., Allam M., Ismail A., Glass A.J., Engelbrecht S. (2021). Sixteen novel lineages of SARS-CoV-2 in South Africa. Nat. Med..

[46] Montagutelli X., Prot M., Levillayer L., Baquero Salazar E., Jouvion G., Conquet L., Donati F., Albert M., Gambaro F., Behillil S., Enouf V., Rousset D., Jaubert J., Rey F., van der Werf S. (2021). The B.1.351 and P.1 variants extend SARS-Cov-2 host range to mice. bioRxiv.

[47] Galloway S.E., Paul P., MacCannell D.R., Johansson M.A., Brooks J.T., MacNeil A., Slayton R.B., Tong S., Silk B.J., Armstrong G.L., Biggerstaff M., Dugan V.G. (2021). Emergence of SARS-CoV-2 B.1.1.7 lineage — United States, December 29, 2020–January 12, 2021. MMWR Morb. Mortal Wkly. Rep..

[48] Ogata A.F., Maley A.M., Wu C., Gilboa T., Norman M., Lazarovits R., Mao C.P., Newton G., Chang M., Nguyen K., Kamkaew M., Zhu Q., Gibson T.E., Ryan E.T., Charles R.C. (2020). Ultra-sensitive serial profiling of SARS-CoV-2 antigens and antibodies in plasma to understand disease progression in COVID-19 patients with severe disease. Clin. Chem..

[49] Goyard S., Balbino B., Chinthrajah R.S., Lyu S.C., Janin Y.L., Bruhns P., Poncet P., Galli S.J., Nadeau K.C., Reber L.L., Rose T. (2020). A highly sensitive bioluminescent method for measuring allergen-specific IgE in microliter samples. Allergy.

[50] Raynal B., Lenormand P., Baron B., Hoos S., England P. (2014). Quality assessment and optimization of purified protein samples: Why and how?. Microb. Cell Fact.

[51] Schuck P. (2000). Size-distribution analysis of macromolecules by sedimentation velocity ultracentrifugation and Lamm equation modeling. Biophys. J..

[52] Lafaye P., Achour I., England P., Duyckaerts C., Rougeon F. (2009). Single-domain antibodies recognize selectively small oligomeric forms of amyloid beta, prevent Abeta-induced neurotoxicity and inhibit fibril formation. Mol. Immunol..

[53] Lescure F.X., Bouadma L., Nguyen D., Parisey M., Wicky P.H., Behillil S., Gaymard A., Bouscambert-Duchamp M., Donati F., Le Hingrat Q., Enouf V., Houhou-Fidouh N., Valette M., Mailles A., Lucet J.C. (2020). Clinical and virological data of the first cases of COVID-19 in Europe: A case series. Lancet Infect. Dis..

[54] Masson G.R., Burke J.E., Ahn N.G., Anand G.S., Borchers C., Brier S., Bou-Assaf G.M., Engen J.R., Englander S.W., Faber J., Garlish R., Griffin P.R., Gross M.L., Guttman M., Hamuro Y. (2019). Recommendations for performing, interpreting and reporting hydrogen deuterium exchange mass spectrometry (HDX-MS) experiments. Nat. Methods.

[55] Yu I.M., Gustafson C.L., Diao J., Burgner J.W., Li Z., Zhang J., Chen J. (2005). Recombinant severe acute respiratory syndrome (SARS) coronavirus nucleocapsid protein forms a dimer through its C-terminal domain. J. Biol. Chem..

[56] Hourdel V., Volant S., O'Brien D.P., Chenal A., Chamot-Rooke J., Dillies M.A., Brier S. (2016). MEMHDX: An interactive tool to expedite the statistical validation and visualization of large HDX-MS datasets. Bioinformatics.

