# Supplementary material for: Development of a highly specific and sensitive VHH-based sandwich immunoassay for the detection of the SARS-CoV-2 nucleoprotein
Source: J Biol Chem. 2021 Oct 20;298(1):101290. doi: 10.1016/j.jbc.2021.101290 (PMC8526496; doi:10.1016/j.jbc.2021.101290)
Supplement: Table S1 and Figs. S1-S9 [file mmc1.docx]

**Development of a highly specific and sensitive VHH-based sandwich immunoassay for the detection of the SARS-CoV-2 nucleoprotein**

**Gransagne M, Aymé A et al**

**This article contains supporting information**

**Figure S1 : Comparison of the binding of VHHs on SARS-CoV-1 and SARS-CoV-2 Nucleoproteins.** An ELISA was performed using the VHHs diluted at different concentrations on coated recombinant Nucleoproteins from seasonal coronaviruses (OC43, HKU1, 229E and NL63), SARS-CoV-1 or SARS-CoV-2 and the Spike protein of SARS-CoV-2 was used as control. The signal corresponding to 50% of the maximal OD measured was used as a reference and the concentration of VHH needed to reach this OD was determined. We represented here the inverse of those concentrations, thus a high value corresponds to a low concentration to achieve 50% of the maximal OD.

**Figure S2: Competition experiments  between different VHHs.** N was captured non-covalently on two flowcells of an NiCl2 loaded NTA sensor chip, and one of them was saturated with VHH E7-2 (100nM). Four VHHs at saturating concentration (100nM for G9-1 and H3-3 ; 1µM for NTD B6-1 and NTD E4-3) were then injected over N and N/E7-2, showing that the epitopes of G9-1 and H3-3 overlap with that of E7-2, while those of NTD B6-1 and NTD E4-3 are distinct.

**Figure S3 | Peptide Map of full-length SARS-CoV-2 N.** The peptide map was generated after 2 min digestion at 20°C with immobilized pig pepsin. Each blue bar corresponds to a unique peptide identified by MS/MS. A total of 51 peptides (blue bar) covering 94.4% of the protein sequence with a 2.43 redundancy value were used to extract HDX data.


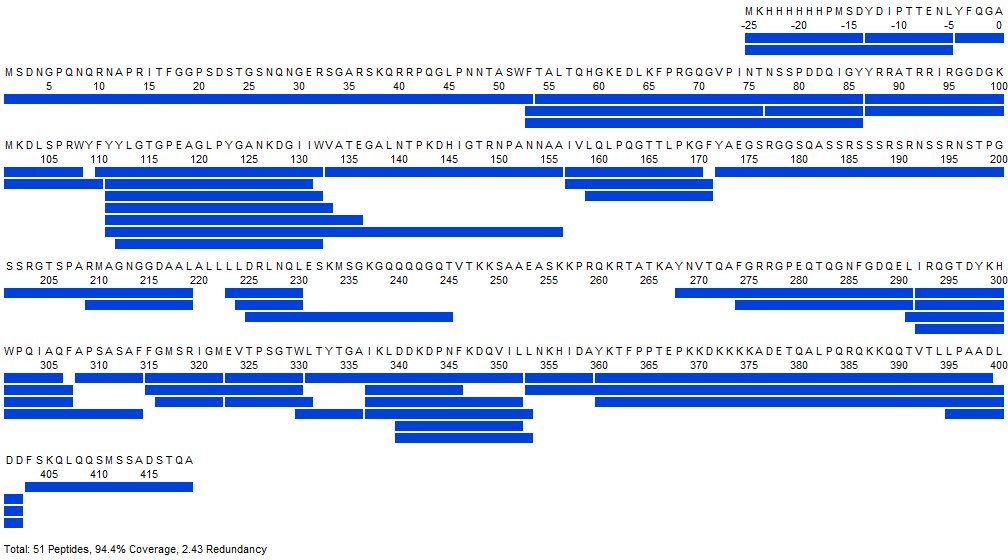


**Figure S4 | Uptake plots for all individual SARS-CoV-2 N peptides generated in the absence (Apo) and presence of VHHs.** Only one charge state was selected per peptide to extract the relative uptakes values. The last time point in the apo state corresponds to the fully deuterated control obtained after 21h incubation at room temperature in deuterated PBS 1X buffer, pD 7.4, and 7.1M final urea-d4.


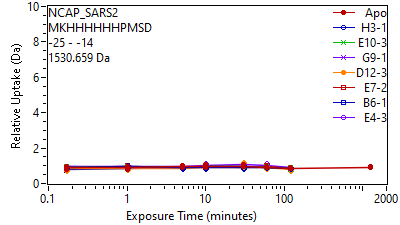

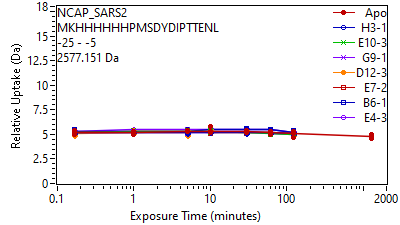

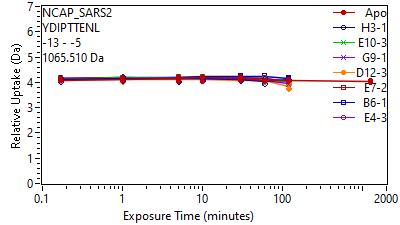

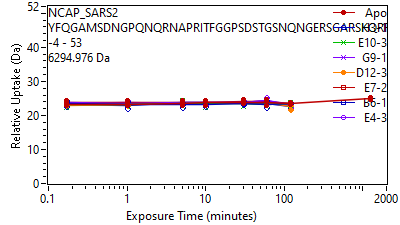

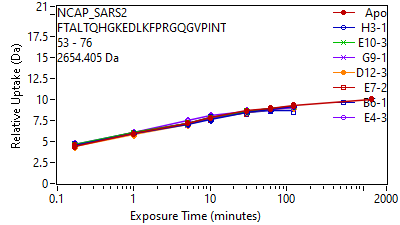

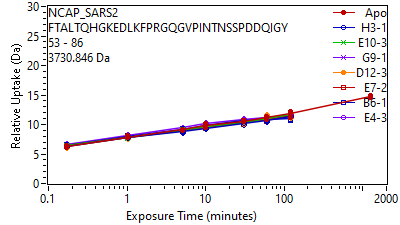


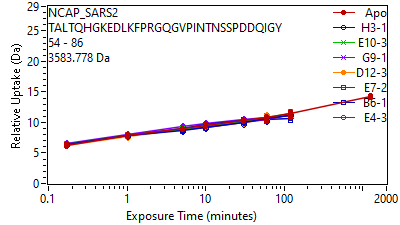

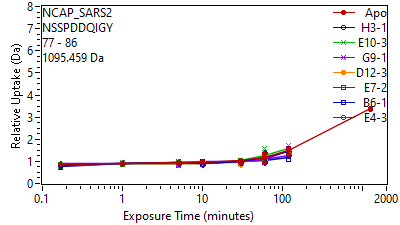

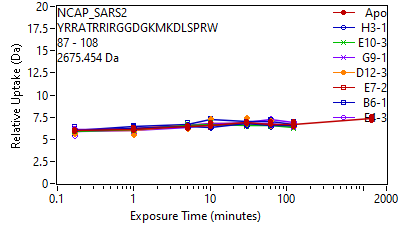

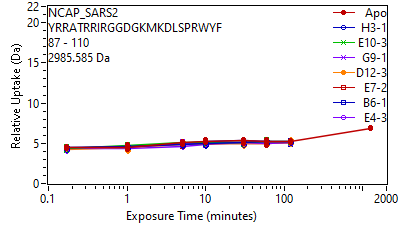

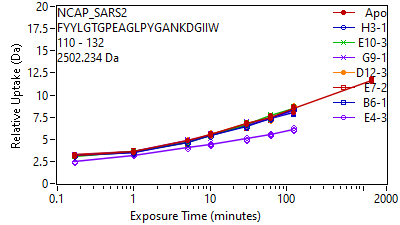

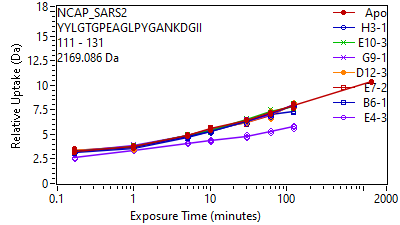

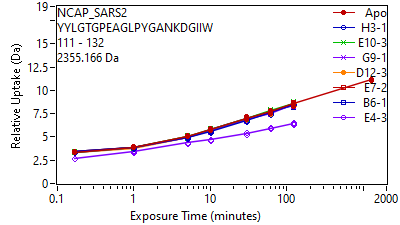

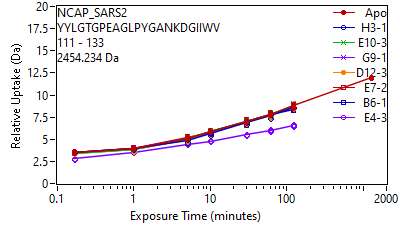

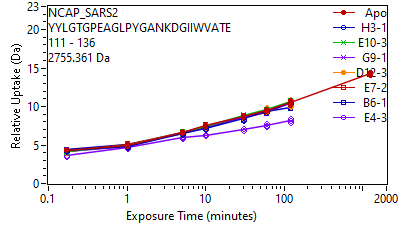

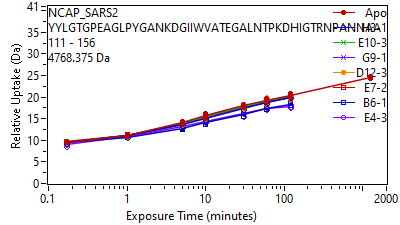

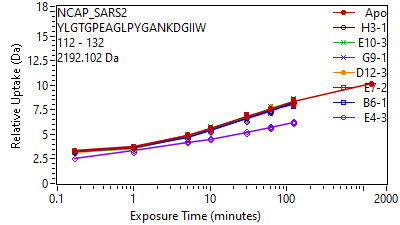

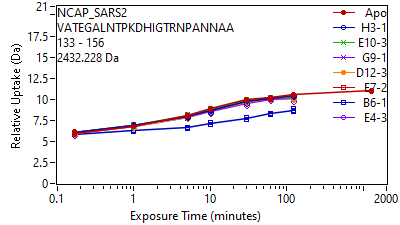


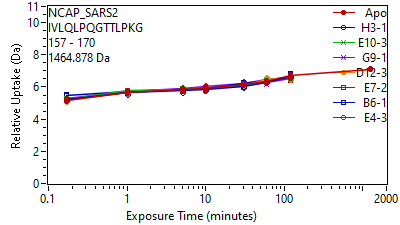

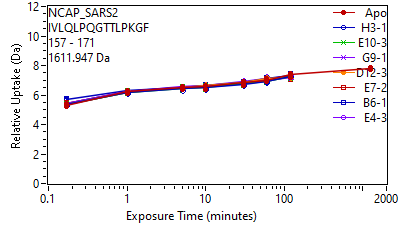

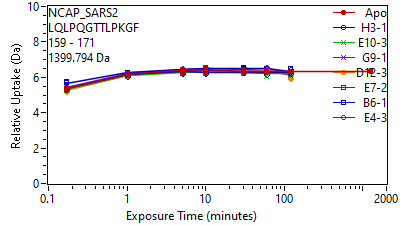


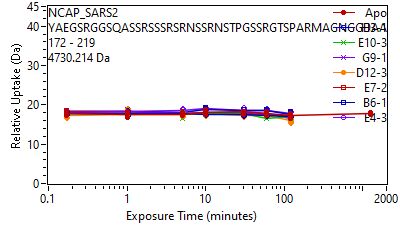

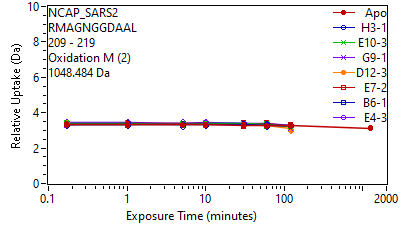

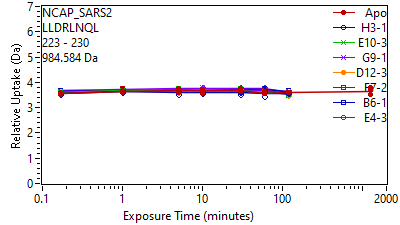

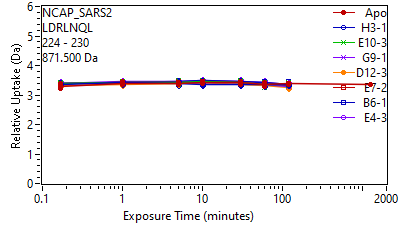

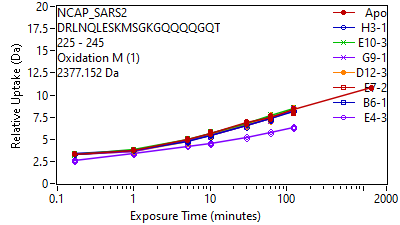

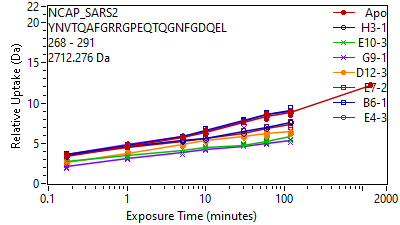

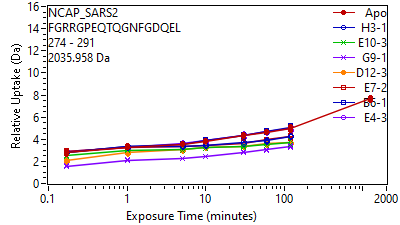

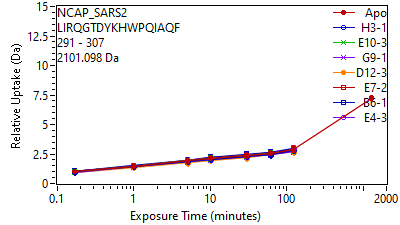

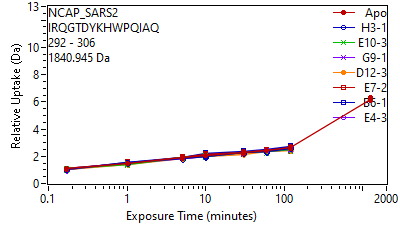


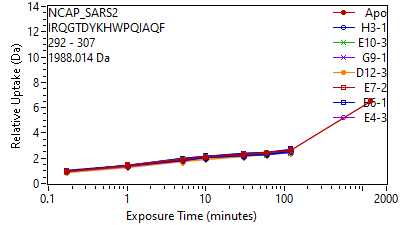

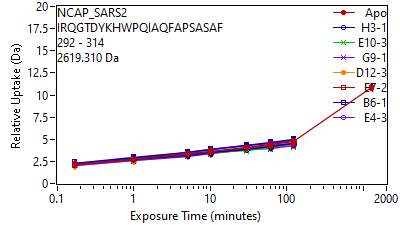

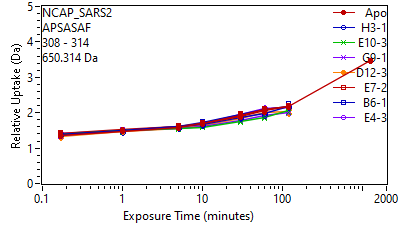


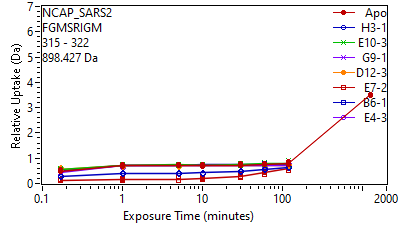

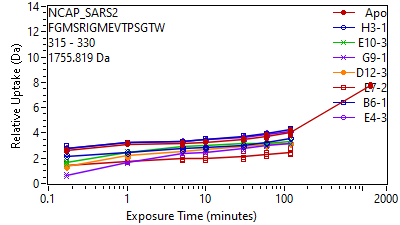

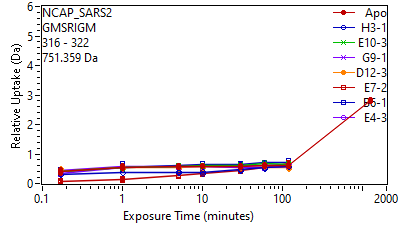


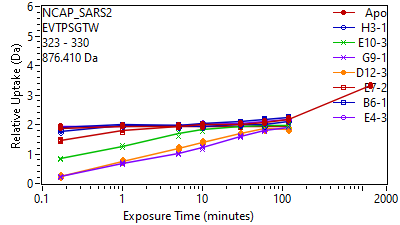

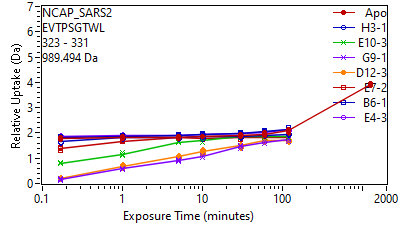

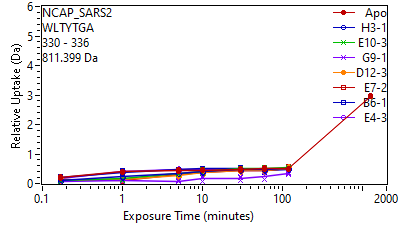


Figure S4 (continued)


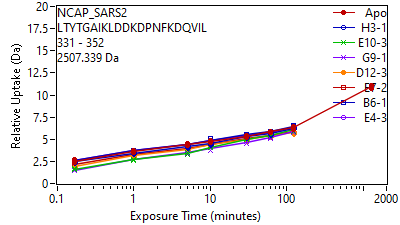

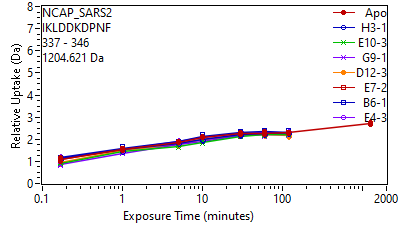

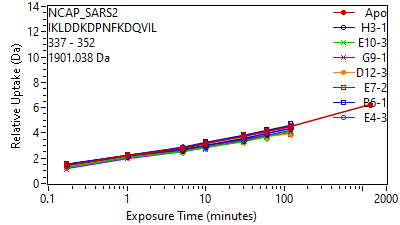


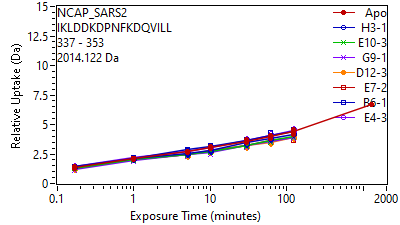

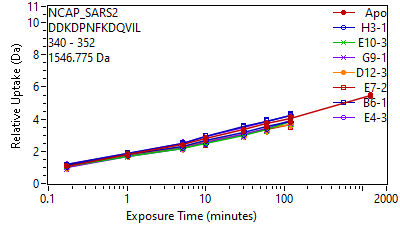

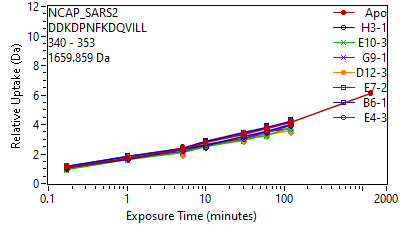

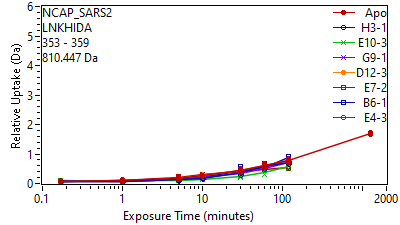

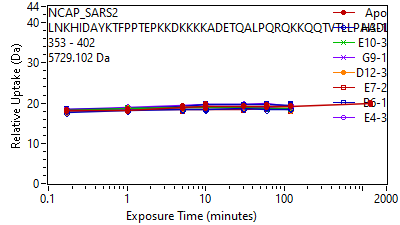

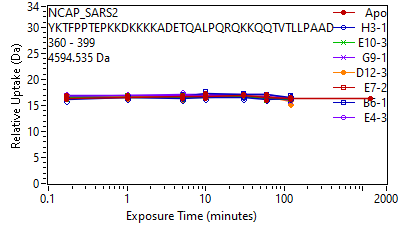


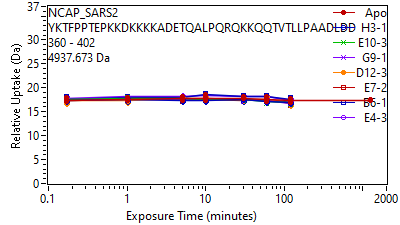

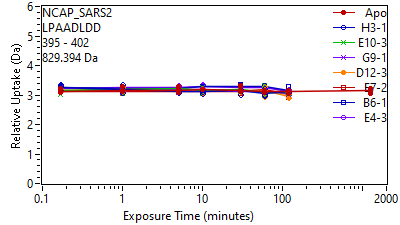

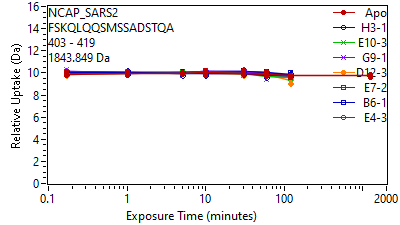


Figure S4 (end)

**Figure S5 | Statistical analysis performed with MEMHDX.** Logit representations of the statistical results obtained for all individual SARS-CoV-2 N peptides in the presence of each VHH. The FDR value was set to 0.05 (red lines). Statistically significant peptides are labeled. Peptides showing no statistically significant differences between states are displayed on the right hand corner of each plot. Statistically significant peptides with Fractional Uptake Difference values below the biological threshold (sets to 3%) are colored in orange (these peptides are not considered as statistically significant; see released note #2 on the MEMHDX website ([www.c3bi.pasteur.fr)](http://www.c3bi.pasteur.fr))).

**Figure S6:** **Detection of Nucleoprotein by sandwich ELISA.** VHH NTD-E4-3 or NTD-B6-1 were coated on the plate, Nucleoproteins from SARS-CoV-2 (**A**) or permeabilized SARS-CoV-2 virus (**B**) were then added at different concentrations and were revealed by adding a biotinylated VHH followed by peroxydase labelled streptavidin. Controls without N or virus were performed and their values were substracted from the data.

**Figure S7: Immunochromatographic assay for qualitative detection of SARS-CoV-2 nucleoprotein**. A: human nasal swabs, each samples are diluted 1/3 in PBS-BSA 1%, B: recombinant Nucleoprotein diluted in PBS-BSA 1%

*
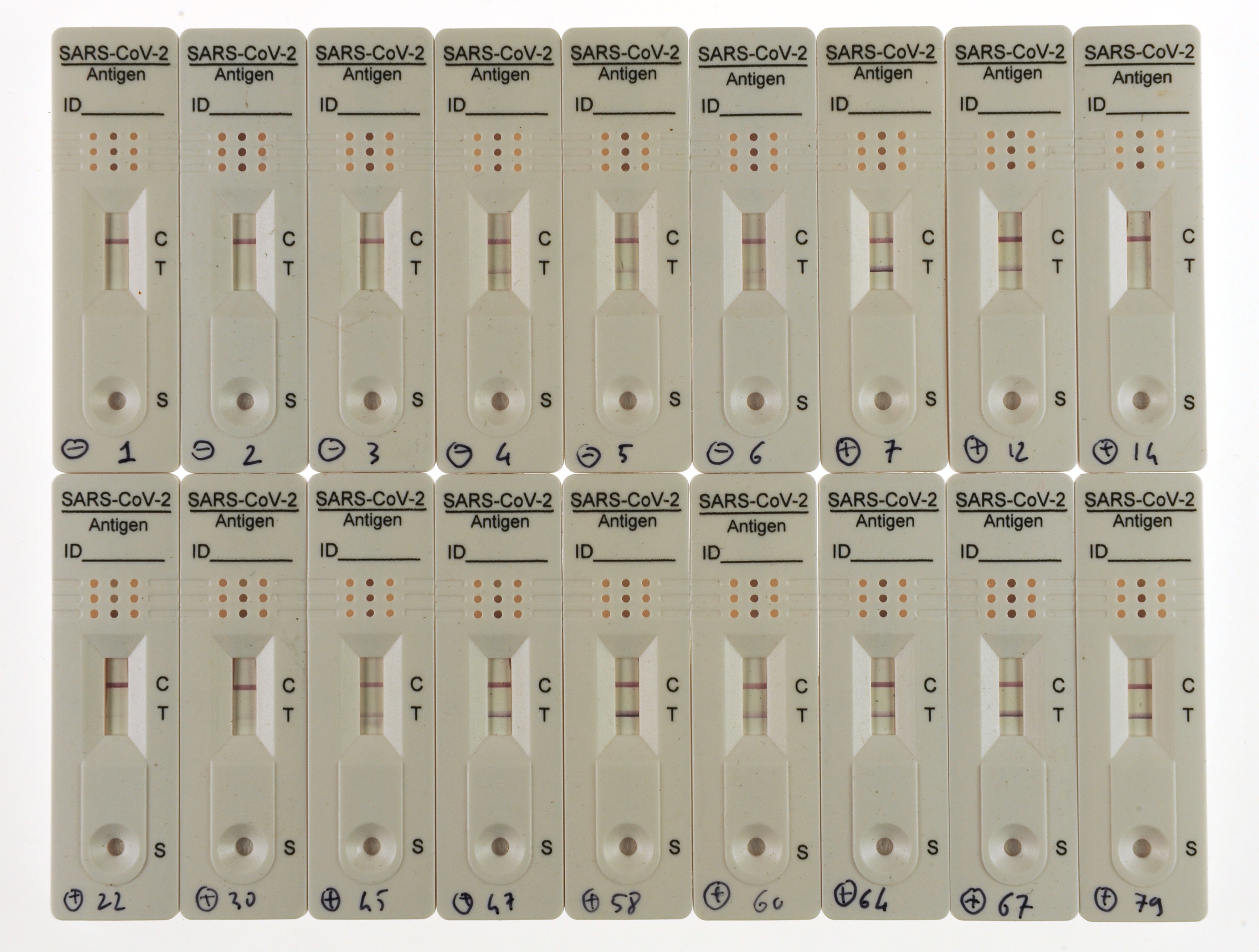
*


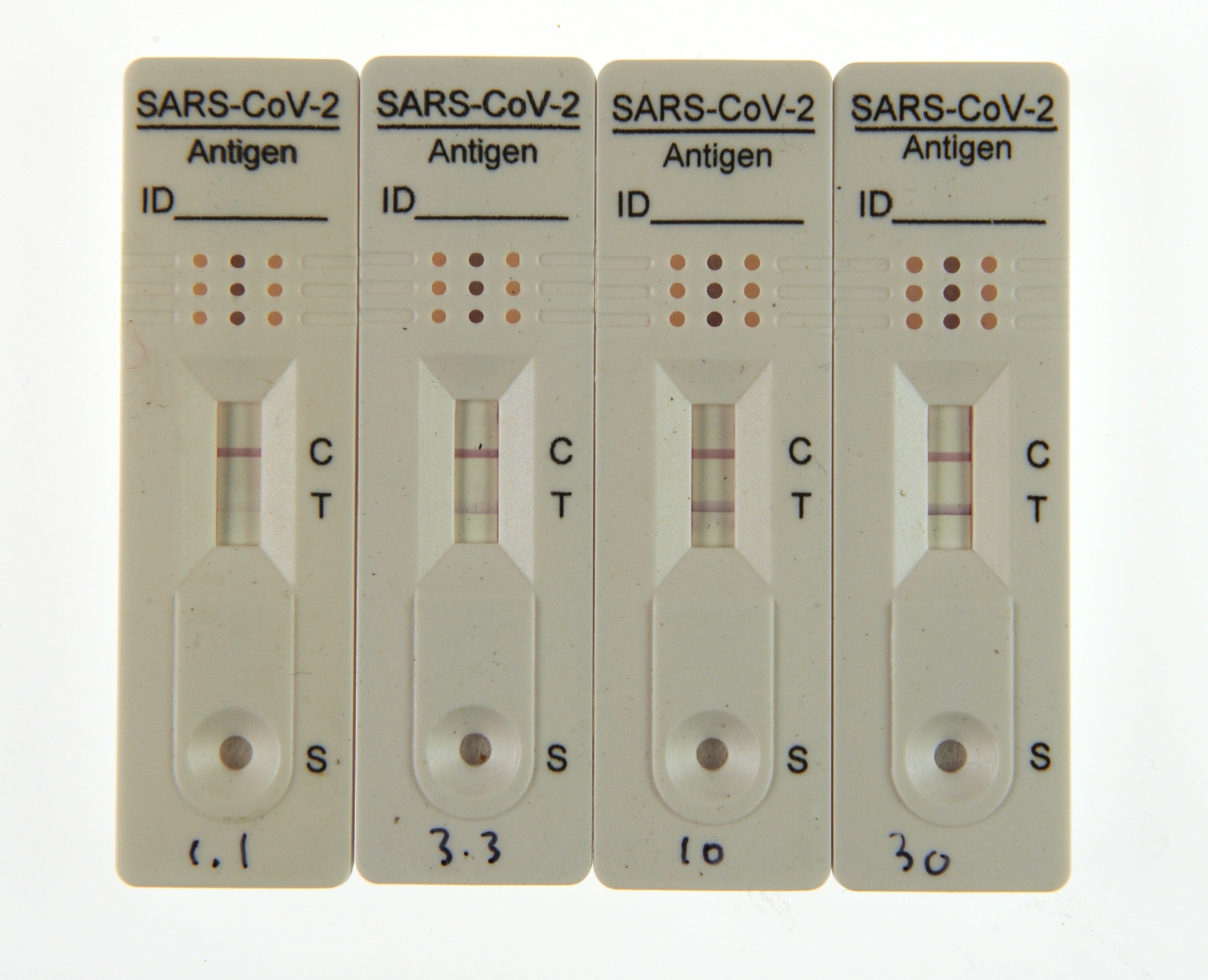


**Figure S8: Detection of Nucleoprotein by sandwich ELISA**. VHH NTD-E4-3 were coated on the plate, and nasal swabs diluted 1/3 were added followed by the addition of biotinylated VHH G9-1 (A). In parallel a calibration curve was performed with recombinant N (B).

**A)**

**B)**

**Figure S9: Alignment of the aminoacid sequences of the nucleoproteins of 229E, NL63, OC43, HKU1, SARS-CoV-1 and SARS-CoV-2.** The different proteins are identified by their UniProt identifier. The alignment was performed by using the Clustal Omega software <https://www.ebi.ac.uk/Tools/msa/clustalo/>. The epitope recognized by VHH E4-3 is in blue, the B6-1 epitope is in green. The epitopic regions recognized by anti-CTD VHHs are in purple. The differences between the SARS N epitopes are highlighted in yellow.

|P15130|-N-229E ---------------------MATVKWADASEPQ-----RGRQG---------------- 18

|Q6Q1R8|-N-NL63 ---------------------MASVNWADDRAA--------RKK---------------- 15

|P59595|-N-SARS1 MSD-NGPQSNQR--------SAPRITFGGPTDSTDNNQNGGRNG----ARPKQRRPQGLP 47

|P0DTC9|-N-SARS2 MSD-NGPQ-NQR--------NAPRITFGGPSDSTGSNQNGERSG----ARSKQRRPQGLP 46

|P33469|-N-0C43 MSFTPGKQSSSRASSGNRSGNG-ILKWADQSDQVRNVQTRGRRAQPKQTATSQQPSGGNV 59

|Q5MQC6|-N-HKU1 MSYTPGHYAGSRSSSGNRSGILKKTSWADQSERNYQTFNRGRKTQPKFTVST--QPQGNT 58

|P15130|-N-229E RIPYSLYSPLLVDS-EQPWKVIPRNLVPINKKD-KNKLIGYWNVQK--RFRTRKGKRVDL 74

|Q6Q1R8|-N-NL63 FPPPSFYMPLLVSSDKAPYRVIPRNLVPIGKGN-KDEQIGYWNVQE--RWRMRRGQRVDL 72

|P59595|-N-SARS1 NNTASWFTALTQHGK-EELRFPRGQGVPINTNSGPDDQIGYYRRAT-RRVRGGDGKMKEL 105

|P0DTC9|-N-SARS2 NNTASWFTALTQHGK-EDLKFPRGQGVPINTNSSPDDQIGYYRRAT-RRIRGGDGKMKDL 104

|P33469|-N-0C43 VPYYSWFSGITQFQKGKEFEFVEGQGPPIAPGVPATEAKGYWYRHNRGSFKTADGNQRQL 119

|Q5MQC6|-N-HKU1 IPHYSWFSGITQFQKGRDFKFSDGQGVPIAFGVPPSEAKGYWYRHSRRSFKTADGQQKQL 118

|P15130|-N-229E SPKLHFYYLGTGPHKDAKFRERVEGVVWVAVDGAKTEPTG-YGVRRKNSEPEIPHFNQKL 133

|Q6Q1R8|-N-NL63 PPKVHFYYLGTGPHKDLKFRQRSDGVVWVAKEGAKTVNTS-LGNRKRNQKPLEPKFSIAL 131

|P59595|-N-SARS1 SPRWYFYYLGTGPEASLPYGANKEGIVWVATEGALNTPKDHIGTRNPNNNAATV---LQL 162

|P0DTC9|-N-SARS2 SPRWYFYYLGTGPEAGLPYGANKDGIIWVATEGALNTPKDHIGTRNPANNAAIV---LQL 161

|P33469|-N-0C43 LPRWYFYYLGTGPHAKDQYGTDIDGVYWVASNQADVNTPADIVDRDPSSDEAIP---TRF 176

|Q5MQC6|-N-HKU1 LPRWYFYYLGTGPYANASYGESLEGVFWVANHQADTSTPSDVSSRDPTTQEAIP---TRF 175

|P15130|-N-229E PNGVTVVE--EPDSRA-----PSRSQSR--SQSRGRGESKPQSRNPSSDRNHNSQDDIMK 184

|Q6Q1R8|-N-NL63 PPELSVVE--FEDRSNNSSRASSRSSTR--NNSRDSSRSTSRQQSRTRSDSNQSSSDLVA 187

|P59595|-N-SARS1 PQGTTLPKGFYAEGSRGGSQASSRSSSRSRGNSRNS---TPGS-SRGNSPARMASGG--- 215

|P0DTC9|-N-SARS2 PQGTTLPKGFYAEGSRGGSQASSRSSSRSRNSSRNS---TPGS-SRGTSPARMAGNG--- 214

|P33469|-N-0C43 PPGTVLPQGYYIEGSGRSAPN-SRSTSR--TSSRAS---SAGSRSRANSGNRTPTSGVTP 230

|Q5MQC6|-N-HKU1 PPGTILPQGYYVEGSGRSASN-SRPGSR--SQSRGP---NNRSLSRSNSNFRHSDSIVKP 229

|P15130|-N-229E AVAAALKSLGFDKPQEKDKKSAKTGTPKPSRNQSPASSQTSAKSLARSQSSETKEQKHEM 244

|Q6Q1R8|-N-NL63 AVTLALKNLGFDNQSKSPSSS---GTSTPKKPNKPL-------------SQPRADKPSQL 231

|P59595|-N-SARS1 -GETALALLLLDRLNQLESKVSGKGQQQ------------------QGQTVTKKSAAEAS 256

|P0DTC9|-N-SARS2 -GDAALALLLLDRLNQLESKMSGKGQQQ------------------QGQTVTKKSAAEAS 255

|P33469|-N-0C43 DMADQIASLVLAKLGKDAT----K-PQQ------------------VTKHTAKEVRQKIL 267

|Q5MQC6|-N-HKU1 DMADEIANLVLAKLGKDS-----K-PQQ------------------VTKQNAKEIRHKIL 265

|P15130|-N-229E QKPRWKRQPNDDVTSNVTQCFGPRDLDH---NFGSAGVVANGVKAKGYPQFAELVPSTAA 301

|Q6Q1R8|-N-NL63 KKPRWKRVPTRE--ENVIQCFGPRDFNH---NMGDSDLVQNGVDAKGFPQLAELIPNQAA 286

|P59595|-N-SARS1 KKPRQKRTATKQ--YNVTQAFGRRGPEQTQGNFGDQDLIRQGTDYKHWPQIAQFAPSASA 314

|P0DTC9|-N-SARS2 KKPRQKRTATKA--YNVTQAFGRRGPEQTQGNFGDQELIRQGTDYKHWPQIAQFAPSASA 313

|P33469|-N-0C43 NKPRQKRSPNKQ--CTVQQCFGKRGPNQ---NFGGGEMLKLGTSDPQFPILAELAPTAGA 322

|Q5MQC6|-N-HKU1 TKPRQKRTPNKH--CNVQQCFGKRGPSQ---NFGNAEMLKLGTNDPQFPILAELAPTPGA 320

|P15130|-N-229E MLFDSHIVSKESG-----------NTVVLTFTTRVTVPKDHPHLGK----FLEELNAFTR 346

|Q6Q1R8|-N-NL63 LFFDSEVSTDEVG-----------DNVQITYTYKMLVAKDNKNLPK----FIEQISAFTK 331

|P59595|-N-SARS1 FFGMSRIGMEVTP-----------SGTWLTYHGAIKLDDKDPQFKDNVILLNKHIDAYKT 363

|P0DTC9|-N-SARS2 FFGMSRIGMEVTP-----------SGTWLTYTGAIKLDDKDPNFKDQVILLNKHIDAYKT 362

|P33469|-N-0C43 FFFGSRLELAKVQNLSGNPDEPQKDVYELRYNGAIRFDSTLSGFETIMKVLNENLNAYQQ 382

|Q5MQC6|-N-HKU1 FFFGSKLDLVKRD---SEADSPVKDVFELHYSGSIRFDSTLPGFETIMKVLEENLNAYVN 377

|P15130|-N-229E EMQQHP-------LLNPSALEF----------NP--SQTSPATAEPVRDEVSIETD---- 383

|Q6Q1R8|-N-NL63 PSSIKE-------MQSQSSHVA----------QNTVLNASIPESKPLADDDSAIIE---- 370

|P59595|-N-SARS1 FPPTEPKKDKKKKTDEAQPLPQRQKKQPTV-------TLL-PAA--DMDDFSRQ------ 407

|P0DTC9|-N-SARS2 FPPTEPKKDKKKKADETQALPQRQKKQQTV-------TLL-PAA--DLDDFSKQ------ 406

|P33469|-N-0C43 QDG----------MMNMSPKPQRQRGHKNGQGENDNISVAVPKSR-VQQNKSRELTAEDI 431

|Q5MQC6|-N-HKU1 SNQNTD-------SDSLSSKPQRKRGVKQLPEQFDSLNLSA-----GTQHISNDFTPEDH 425

|P15130|-N-229E ----IID--------EVN------ 389

|Q6Q1R8|-N-NL63 ----IVN--------EVLH----- 377

|P59595|-N-SARS1 -LQNSMSGASADSTQA-------- 422

|P0DTC9|-N-SARS2 -LQQSMSSADSTQA---------- 421

|P33469|-N-0C43 SLLKKMDEPYTEDTSEI------- 448

|Q5MQC6|-N-HKU1 SLLATLDDPYVEDSVA-------- 44

**Supplementary Table S1:** HDX summary data

| **DATA SETS** | **Apo**  **State** | **SARS-CoV-2 nucleoprotein + VHH** | | | | | | |
| --- | --- | --- | --- | --- | --- | --- | --- | --- |
|  |  | **NTD B6-1** | **D12-3** | **NTD E4-3** | **E7-2** | **E10-3** | **G9-1** | **H3-3** |
| HDX reaction details:  *- pD*  *- T°C*  *- Deuterium level (%)*  *+ during labeling*  *+ after quench*  *- VHH molar excess*  *- Kd (nM)*  *- % complex during labeling ^&^* | 7.4  RT^(a)^  88.6  14.8  /  /  / | 7.4  RT  88.6  14.8  ~1.2  46.5  89 | 7.4  RT  88.6  14.8  ~1.3  7.0  98 | 7.4  RT  88.6  14.8  ~1.4  23.8  96 | 7.4  RT  88.6  14.8  ~1.2  0.17  99 | 7.4  RT  88.6  14.8  ~1.3  24.1  95 | 7.4  RT  88.6  14.8  ~1.2  2.40  99 | 7.4  RT  88.6  14.8  ~1.1  3.99  98 |
| HDX time course analyzed (min) | 0.16, 1, 5, 10, 30, 60, and 120 | | | | | | | |
| HDX control  Average Back Exchange | Fully-labeled control (21h @ RT, 88.4% D_2_0, PBS 1X, 8M urea d4-0, pD 7.4)  **42%** (Apo state, 3 independent technical replicates) | | | | | | | |
| Sequence coverage  Number of peptides  Average peptide length  Redundancy | 94.4%  51  20.04  2.43 | 94.4%  51  20.04  2.43 | 94.4%  51  20.04  2.43 | 94.4%  51  20.04  2.43 | 94.4%  51  20.04  2.43 | 94.4%  51  20.04  2.43 | 94.4%  51  20.04  2.43 | 94.4%  51  20.04  2.43 |
| Average peptide length / Redundancy Ratio | 8.24 | 8.24 | 8.24 | 8.24 | 8.24 | 8.24 | 8.24 | 8.24 |
|  |  |  |  |  |  |  |  |  |
| Replicates (technical) | 3 | 3 | 3 | 3 | 3 | 3 | 3 | 3 |
| Repeatability (pooled standard deviation, Da) # | 0.098 | 0.089 | 0.080 | 0.099 | 0.112 | 0.112 | 0.088 | 0.094 |
| Significance difference between state * | Wald test, *p*< 0.01; Biological threshold sets to 3% | | | | | | | |

^&^ considering a 1 to 1 binding stoichiometry between the nucleocapsid monomer and each VHH

# one unique charge state was selected per peptide

* MEMHDX ([www.c3bi.pasteur.fr](http://www.c3bi.pasteur.fr))

^(a)^ room temperature
